# New insights into the role of mitochondrial metabolic dysregulation and immune infiltration in septic cardiomyopathy by integrated bioinformatics analysis and experimental validation

**DOI:** 10.1186/s11658-024-00536-2

**Published:** 2024-01-30

**Authors:** Yukun Li, Jiachi Yu, Ruibing Li, Hao Zhou, Xing Chang

**Affiliations:** 1 Department of Cardiology, Beijing Anzhen Hospital, Capital Medical University, Beijing, China; 2 National Clinical Research Center for Cardiovascular Diseases, Beijing, China; 3 Department of Cardiology, The Sixth Medical Center of People’s Liberation Army General Hospital, Beijing, China; 4 Guanganmen Hospital, China Academy of Chinese Medical Sciences, Beijing, China

**Keywords:** Septic cardiomyopathy, Molecular mechanism, Drug discovery, Mitochondrial metabolism, Immune infiltration

## Abstract

**Background:**

Septic cardiomyopathy (SCM), a common cardiovascular comorbidity of sepsis, has emerged among the leading causes of death in patients with sepsis. SCM’s pathogenesis is strongly affected by mitochondrial metabolic dysregulation and immune infiltration disorder. However, the specific mechanisms and their intricate interactions in SCM remain unclear. This study employed bioinformatics analysis and drug discovery approaches to identify the regulatory molecules, distinct functions, and underlying interactions of mitochondrial metabolism and immune microenvironment, along with potential interventional strategies in SCM.

**Methods:**

GSE79962, GSE171546, and GSE167363 datasets were obtained from the Gene Expression Omnibus (GEO) database. Differentially expressed genes (DEGs) and module genes were identified using Limma and Weighted Correlation Network Analysis (WGCNA), followed by functional enrichment analysis. Machine learning algorithms, including support vector machine–recursive feature elimination (SVM–RFE), least absolute shrinkage and selection operator (LASSO) regression, and random forest, were used to screen mitochondria-related hub genes for early diagnosis of SCM. Subsequently, a nomogram was developed based on six hub genes. The immunological landscape was evaluated by single-sample gene set enrichment analysis (ssGSEA). We also explored the expression pattern of hub genes and distribution of mitochondria/inflammation-related pathways in UMAP plots of single-cell dataset. Potential drugs were explored using the Drug Signatures Database (DSigDB). In vivo and in vitro experiments were performed to validate the pathogenetic mechanism of SCM and the therapeutic efficacy of candidate drugs.

**Results:**

Six hub mitochondria-related DEGs [MitoDEGs; translocase of inner mitochondrial membrane domain-containing 1 (TIMMDC1), mitochondrial ribosomal protein S31 (MRPS31), F-box only protein 7 (FBXO7), phosphatidylglycerophosphate synthase 1 (PGS1), LYR motif containing 7 (LYRM7), and mitochondrial chaperone BCS1 (BCS1L)] were identified. The diagnostic nomogram model based on the six hub genes demonstrated high reliability and validity in both the training and validation sets. The immunological microenvironment differed between SCM and control groups. The Spearman correlation analysis revealed that hub MitoDEGs were significantly associated with the infiltration of immune cells. Upregulated hub genes showed remarkably high expression in the naive/memory B cell, CD14^+^ monocyte, and plasma cell subgroup, evidenced by the feature plot. The distribution of mitochondria/inflammation-related pathways varied across subgroups among control and SCM individuals. Metformin was predicted to be the most promising drug with the highest combined score. Its efficacy in restoring mitochondrial function and suppressing inflammatory responses has also been validated.

**Conclusions:**

This study presents a comprehensive mitochondrial metabolism and immune infiltration landscape in SCM, providing a potential novel direction for the pathogenesis and medical intervention of SCM.

**Supplementary Information:**

The online version contains supplementary material available at 10.1186/s11658-024-00536-2.

## Introduction

Due to its high morbidity, sepsis remains one of the leading causes of death worldwide, making it a pressing public health issue [1]. The third international consensus definition for sepsis (sepsis-3) given in 2016 suggests that sepsis is a life-threatening organ dysfunction caused by a dysregulated host response to infection [2]. Given that the heart is among the most vulnerable organs to sepsis, the incidence rate of complication with myocardial injury in patients with sepsis ranges from 13.8% to 40%, with an astonishingly high mortality rate of 70–90%, severely threatening human health [3]. Septic cardiomyopathy (SCM) was first reported more than 40 years ago [4]. It is a kind of acute but reversible cardiac disease caused by sepsis, with a window of opportunity for recovery in the early stages [5]. Multiple genes and complex phenotypes contribute to the pathogenesis of SCM [6, 7]. Following diagnosis, the current recommended strategy for SCM is symptomatic treatment [3, 8]. Thus, for discovering novel therapeutic targets and strategies for prediction in the early stages, a better understanding of the pathogenesis of SCM is necessitated. Identifying hub genes associated with SCM is essential for the early detection, prevention, and management of SCM.

SCM is primarily characterized by myocardial damage mediated by immune infiltration. However, preclinical and clinical studies have demonstrated limited efficacy of anti-inflammatory strategies in reducing SCM-related mortality [9, 10], suggesting that the pathogenesis of SCM involves the interaction between inflammation and some other mechanisms. The key characteristic of SCM is left ventricular systolic and diastolic dysfunction. Contraction and relaxation of the myocardium are extremely energy-intensive processes, relying heavily on the supply of adenosine triphosphate (ATP) from mitochondria. Previous studies from our group have highlighted mitochondrial metabolic disorders in SCM, including abnormal mitochondrial dynamic balance, dysfunctional mitophagy, and defective mitochondrial bioenergetics [11–16]. These results are indicative of a causal relationship between mitochondrial dysfunction and SCM, and targeting mitochondria may facilitate early detection and appropriate treatment of SCM.

Accumulating evidence suggests a potential link between immune disorder and mitochondrial dysfunction. Upon disruption of mitochondrial homeostasis, several mitochondrial components and metabolic by-products leak out of organelles. Consequently, they can function as damage-associated molecular patterns (DAMPs) that trigger inflammation upon release into the cytosol or extracellular environment [17–19]. Inflammatory cytokines including TNFα or IL-1β can activate inflammatory and oxidative stress pathways in the mitochondrial membrane system, causing oxidative damage to mitochondrial DNA and membrane phospholipids, thereby disrupting the physiological mitochondrial energy metabolism. These cytokines can induce mitochondrial permeability transition pore (mPTP) opening and mitochondrial outer membrane permeabilization, both of which can eventually result in cell death [20–23].

Taken together, mitochondrial dysfunction and immune disorder are essential pathogenic factors for the development of SCM. However, reports on these issues are sporadic and require further in-depth investigation. The field of biomedical research has advanced substantially owing to the development of high-throughput genomics. By identifying differentially expressed genes (DEGs) between healthy individuals and patients with SCM, high-throughput sequencing provides a comprehensive overview of the alterations in mitochondrial metabolism and inflammatory pathways during the pathogenesis of SCM and elucidates the interaction between these biological pathways along with the underlying molecular mechanisms. Machine learning has also been employed to identify hub biomarkers of interest for the diagnosis of SCM. Herein, we integrated bioinformatics analysis and machine learning approaches to elucidate the involvement of mitochondria and immune infiltration in the processes of SCM based on microarray and single-cell RNA-sequencing data sourced from the GEO database. The association between hub mitochondria-related genes and immune infiltration in SCM was investigated to provide insight into the underlying immunometabolic interplay during disease progression.

## Methods

### Retrieving expression profiles

For a comprehensive retrieval of SCM datasets, the NCBI Gene Expression Omnibus database (http://www.ncbi.nlm.nih.gov/geo), a primary source for high-throughput genomic data, was queried. Our search strategy involved terms including “septic cardiomyopathy,” “sepsis,” and “septic heart,” ensuring a broad, yet specific, dataset retrieval. Postretrieval, datasets were meticulously screened based on the study type (array/high throughput sequencing) and species (*Homo sapiens*/*Mus musculus*), following the established protocols. Finally, GSE79962, GSE171546, and GSE167363 datasets were included.

The GSE79962 dataset, generated on the GPL6244 platform, was integral to our analysis. This dataset comprised 51 left ventricular tissue samples from diverse patient groups. Specifically, our study focused on 20 patients with sepsis and 11 healthy donors as controls, resulting in a total of 31 samples for in-depth analysis of DEGs between the SCM and control groups. The patient group comprised 20 individuals who succumbed to systemic sepsis in surgical/medical intensive care units at the Barnes-Jewish Hospital between 2009 and 2012. The mean age of these patients was 70 ± 3 years, with a balanced sex distribution of 10 males and 10 females. The primary sites of infection in these patients included the gastrointestinal system (18 cases), pulmonary system (8 cases), urinary tract (2 cases), and necrotizing fasciitis (2 cases). The average length of hospital stay was 9 days, with a median length of ICU stay of 9 days and a median sepsis duration of 3 days. Pertinent comorbidities in these patients included hypertension (14 cases), type II diabetes mellitus (5 cases), coronary artery disease (4 cases), and others. The microbial etiology of patients with sepsis in this dataset was diverse, comprising those with Gram-positive (two cases), Gram-negative (seven cases), and fungal (one case) infections. This variety in sepsis types can provide a comprehensive perspective for analyzing the pathophysiology of SCM and its differential expression in the context of varying microbial assaults. Moreover, GSE171546, generated from the GPL24247 platform, included 20 myocardial samples from SCM and control mice. The single-cell RNA-sequencing (RNA-seq) dataset, GSE167363, based on the GPL24676 platform, comprised human peripheral blood mononuclear cells (PBMCs) from sepsis survivors and nonsepsis donors, thereby providing a unique perspective on SCM at the single-cell level.

### Acquisition of microarray data and identification of DEGs

Microarray data from the GEO database were systematically retrieved and processed using the “GEOquery” package (version 2.66.0), a robust tool for handling GEO datasets [24]. DEG analysis was conducted using the “limma” package (version 3.54.0), a widely accepted method for analyzing data on gene expression [25]. The “ggplot2” (version 3.4.0) and “ComplexHeatmap” (version 2.14.0) packages were used for the visualization and generation of volcano plots and heatmaps, respectively, to facilitate an intuitive understanding of the data.

### Functional enrichment analysis

Gene Set Enrichment Analysis (GSEA), a powerful tool for interpreting gene expression data, was performed using the “clusterProfiler” package (version 4.2.2), renowned for its comprehensive analysis of functional profiles [26]. We utilized the “c2.cp.v7.2.symbols.gmt” gene set from the Molecular Signatures Database (MSigDB) (https://www.gsea-msigdb.org/gsea/msigdb/index.jsp) with a stringent permutation count of 10,000 to ensure robust statistical power. The significance threshold was set at *p* < 0.10. Results of GSEA were visualized using the “ggplot2” package for an intuitive understanding of the data.

We performed comprehensive functional enrichment analyses to elucidate the potential functions of the identified targets. Gene Ontology (GO) analysis, including molecular functions (MF), biological pathways (BP), and cellular components (CC), was performed for functional annotation of genes. A Kyoto Encyclopedia of Genes and Genomes (KEGG) enrichment analysis was performed to integrate gene functions with high-level genomic functional data to enhance the understanding of the roles of SCM-related target genes. These analyses were performed using the “clusterProfiler” and “GOplot” packages (version 1.0.2), which are widely recognized for their efficacy in functional analysis.

### Weighted gene coexpression network analysis (WGCNA)

In this study, WGCNA was employed to construct a network of gene modules for identifying clusters of highly correlated genes. This approach, as detailed by Langfelder and Horvath, allows the elucidation of correlational patterns among genes across different samples, thereby providing insights into the underlying biological mechanisms [27].

Initially, we conducted sample clustering to detect and exclude outliers, thus ensuring the robustness of our network analysis. The network construction was then performed using a soft-thresholding power to emphasize strong correlations and penalize weaker ones, a key feature of WGCNA that enhances network specificity and sensitivity. This step was followed by the identification of gene modules through hierarchical clustering, using the dynamic tree-cutting method. Each module was represented by a specific color for easy visualization and interpretation. We calculated both module membership (MM) and gene significance (GS) to determine the association of these modules to clinical traits of SCM. MM quantifies the correlation of each gene with a given module, while GS estimates the correlation of genes with external clinical traits. Modules with the highest correlation and significant GS (MM > 0.8 and GS > 0.2) were considered biologically significant.

Finally, hub genes within these significant modules were identified based on their intramodular connectivity, indicative of their central role in the network. These hub genes were prioritized for further analysis because they likely play key roles in the pathogenesis of SCM.

### Identification of hub genes using the machine learning approach

Identification of hub genes pivotal in SCM was achieved using advanced machine learning techniques. Initially, we employed the support vector machine (SVM) algorithm, a robust supervised learning method, to train a model based on a subset of feature genes. This approach is effective in handling high-dimensional data because it focuses on maximizing the margin between different classes [28]. Subsequently, SVM-recursive feature elimination (SVM–RFE) was performed to iteratively refine the feature set by eliminating the least significant features, thereby enhancing the model’s predictive accuracy. This step was crucial in narrowing down the most informative genes for the diagnosis of SCM.

The feature set was refined further by Least Absolute Shrinkage and Selection Operator (LASSO) regression using the “glmnet” package (version 4.1.8). LASSO regression is known for its ability to perform variable selection and regularization, which helps in preventing model overfitting [29]. The selection of variables in the LASSO model was based on the 1-SE criterion to ensure a balance between model complexity and performance. Lastly, the random forest algorithm was employed to rank the genes based on their importance. This ensemble learning method is effective in handling unbalanced data and provides an estimate of feature importance, which is crucial for identifying key genes. Genes with a relative importance score above 0.25 were considered significant. The final set of hub genes was determined by an intersection analysis of the results obtained from LASSO logistic regression, SVM-RFE, and random forest methods, ensuring a robust and comprehensive selection.

### Nomogram construction and receiver operating characteristic (ROC) curve evaluation

The nomogram, a vital tool for clinical prediction, was constructed with hub genes to enhance the diagnostic accuracy of SCM using the “rms” package (version 6.5–0). In this nomogram, each gene was assigned a specific score (“points”), with the “total points” representing the cumulative score of all included genes. This scoring system translates complex genetic data into a user-friendly graphical representation, thereby aiding clinicians in decision making.

To assess the diagnostic efficacy of the nomogram, we constructed a receiver operating characteristic (ROC) curve. The area under the curve (AUC) and the corresponding 95% confidence interval (CI) were calculated to quantify the diagnostic performance, thus providing a measure of the model’s ability to distinguish between the SCM and control groups. The AUCs were calculated using the “pROC” package (version 1.18.5) in R, which is a tool that implements a nonparametric approach for estimating the AUC. This method is particularly advantageous, as it does not assume a specific distribution for the data, making it suitable for a wide range of datasets. The “pROC” package employs the DeLong method for calculating the AUC, which is a widely accepted technique for ROC analysis in biomedical research. This method compares the observed ROC curve against a null hypothesis of no discrimination (AUC of 0.5) for accurate and unbiased estimates of the model’s diagnostic ability. The AUC is a measure of the model’s ability to discriminate between positive and negative cases, with values closer to 1 suggesting higher diagnostic accuracy [30]. The predictive utility of the nomogram was externally validated using an independent dataset (GSE171546). This validation involved the construction of ROC curves for the validation set, ensuring the model s robustness and applicability in different sample cohorts.

### Single-sample gene set enrichment analysis (ssGSEA)

In our study, ssGSEA, a method for quantifying gene set enrichment in individual samples, was employed using the “Gene Set Variation Analysis” (GSVA) package in R. This approach allows for the assessment of variations in pathways and biological processes across a sample population, thus providing insights into the heterogeneity of immune responses in patients with SCM [31]. Specifically, ssGSEA was performed to evaluate the infiltration levels of 28 distinct immune cell types and discern alterations in the above gene sets between the SCM and control groups.

The Wilcoxon rank-sum test, a nonparametric test that does not assume a normal distribution of the data, was used to compare results between these groups. To explore the correlation between different types of immune cells, the Spearman correlation analysis was conducted. This nonparametric measure of rank correlation assesses how well the relationship between two variables can be described using a monotonic function to provide a comprehensive view of immune cell interactions in SCM.

### Analysis of single-cell RNA-seq data

We used the single-cell transcriptome dataset (GSE167363) from the GEO database, comprising data from PBMCs of healthy controls and patients with Gram-negative sepsis. Raw data were preprocessed using the Seurat R package (version 4.3.0), a widely recognized tool for the analysis of single-cell genomics data, thus ensuring the accuracy and reliability of our findings [32].

Key metrics including the number of molecules per cell (nCount RNA) and the number of genes detected per cell (nFeature RNA) were determined. These metrics were juxtaposed against the sequencing read counts to ensure data integrity. We assessed mitochondrial genomic contamination, a commonly encountered issue in low-quality or dead cells, by calculating the percentage of reads mapping to the mitochondrial genome using the percentage feature set function in Seurat.

For cell clustering, we employed the dimensionality reduction technique of unified manifold approximation and projection (UMAP), following the filtration of principal components. This approach allowed for a clear visual classification of cell clusters. Statistically significant cell marker genes (adjusted *p*-values < 0.05) were identified and used to determine the class group of clustered cells. This was achieved by cross-referencing cell marker genes from the DISCO database (https://www.immunesinglecell.org/) with genes specific to each class group, providing insights into the distribution and abundance of hub genes across different cell subpopulations.

The ‘irGSEA’ package (https://github.com/chuiqin/irGSEA) was used for refined gene set enrichment analysis in single-cell transcriptomics, focusing on batch effect minimization. Individual cells were scored using methods that are less affected by batch variations, including AUCell, UCell, and modified ssGSEA. This approach generated multiple enrichment score matrices, enabling the identification of DEG sets in each cell subpopulation. The Wilcoxon test was used to determine the significance of the results. Heatmaps, density scatterplots, and ridge plots were used for visualization, highlighting specific enrichment pathways, particularly in exploring mitochondria/inflammation-related pathways in patients with SCM.

### Drug prediction and molecular docking

Predicting protein-drug interactions is crucial in comprehending the structural characteristics recommended for receptor sensitivity. MitoDEGs have been submitted to the Drug Signatures Database (DSigDB, http://dsigdb.tanlab.org/DSigDBv1.0/), containing 22,527 gene clusters relevant for drug prediction. The Enrichr platform provides access to the DSigDB database. Candidate drugs were ranked in the ascending order of their adjusted *p*-values. An adjusted *p*-value < 0.01 was deemed statistically significant.

Small molecules and the aforementioned hub targets were docked using AutoDock Vina (Scripps Research, San Diego, CA). The results of docking were evaluated and analyzed using the PLIP system (https://plip-tool.biotec.tu-dresden.de/plip-web/plip/index). Finally, the molecular docking (MD) outcomes of the two-dimensional structures were visualized using the LIGPLOT software version 4.5.3 (European Bioinformatics Institute, Cambridge, UK), and MD maps were generated using PyMOL. Protein structures were obtained from PDB (https://www.pdb.org/) or AlphaFold (https://alphafold.com/), and data on metformin were retrieved from PubChem (https://pubchem.ncbi.nlm.nih.gov/).

### Animals and echocardiography

Adult male C57BL/6 mice (aged 8–10 weeks and weighing 20–25 g) were obtained from the Beijing Sibeifu Experimental Animal Center in Beijing, China. Experimental procedures of animal studies were conducted in accordance with the ARRIVE guidelines and the Basel Declaration. All animal experiments were approved by the Ethics Committee of Guang'anmen Hospital (approval number: IACUC-GAMH-2023–054-SQ).

To induce septic cardiomyopathy, a single dose of lipopolysaccharide dissolved in phosphate buffer saline (PBS, 20 mg/kg, Sigma) was intraperitoneally injected in mice for 48 h as described in previous studies [14, 33]. Mice that received an equivalent amount of PBS served as the controls. For metformin intervention, mice were administered a single dose of metformin (250 mg/kg, i.p, Sigma) dissolved in saline solution 12 h before the LPS injection. The heart tissue samples were promptly harvested and stored at −80 °C for further analysis. Before conducting echocardiography, the mice were sufficiently anesthetized. Echocardiography was performed with the assistance of the transducer from a high-resolution imaging system. The left ventricular (LV) parameters such as left ventricular ejection fraction (LVEF), fraction shortening (FS), and left ventricular internal diameters measured during left ventricular internal diameter end systole (LVDs) and diastole (LVDd) were determined from the long-/short-axis images of the left ventricle.

### Cell culture

Immortalized mouse cardiac HL-1 cells were obtained from the American Type Culture Collection (ATCC, Manassas, VA). The cells were cultured in Dulbecco’s modified Eagle’s medium (DMEM) supplemented with glutamine and incubated at 37 °C with 5% CO_2_. The culture media were additionally supplemented with 10% fetal bovine serum (FBS) and 100 mg/mL of penicillin and streptomycin combination solution. To simulate SCM in vitro, HL-1 cells were treated with 10 mg/mL of LPS (Sigma) with or without 4 mM of metformin (Sigma) for 24 h, according to previously published studies [14, 34]. To establish the BCS1L knockdown model, HL-1 cells were transfected with siRNA targeting BCS1L (siBCS1L, obtained from Obio Technology Corp, China).

### RNA extraction and quantitative real-time PCR (qRT-PCR)

Total RNA was extracted using TRIzol reagent (T9424, Sigma), which was then purified using the phenol–chloroform method. The cDNA synthesis was performed by reverse transcription of RNA, using Transcriptor First-Strand cDNA Synthesis Kit (04896866001, Roche, Basel, Switzerland). Subsequently, qRT-PCR was conducted using SYBR Green (04887352001, Roche). The PCR amplification protocol comprised an initialization step at 94 °C for 2 min, 40 cycles of denaturation at 94 °C for 30 s, annealing at 45 °C for 30 s, and elongation at 72 °C for 105 s, and a final elongation at 72 °C for 10 min. β-actin was selected as the internal reference gene. The primer sequences were as follows: *FBXO7* (forward, 5′-CCCACGTTGGGGTTCAGTTC-3′ and reverse, 5′-TCCTGGAGTGAGGAATGCTCT-3′), *PGS1* (forward, 5′-CCCACCTTGCTGCCTATGTC-3′ and reverse, 5′-GCCATCACAACTCGCCTCT-3′), *BCS1L* (forward, 5′-TGTTCTGGCCCTTAAAGACAATC-3′ and reverse, 5′-GGAATGCCACTAGACCCAACT-3′), *LYRM7* (forward, 5′-GTCAGCCCGCCAAGGTTTTA-3′ and reverse, 5′-CAGTACGGCACATTTTCTGTGA-3′), *TIMMDC1* (forward, 5′-CGTTCAGGTGTCTCAGACCC-3′ and reverse, 5′-TAAATCTCCGCTTGGCTCTGT-3′), *MRPS31* (forward, 5′-CTCCACAGAATCCCGGCATTT-3′ and reverse, 5′-ACTGGTCAACTTTCTTGCTACAG-3′).

### Analysis of mitochondrial membrane potential

Isolated primary cardiomyocytes were incubated with the mitochondrial potential sensor JC-1 (10 nM; T3168, Invitrogen) in the dark for 15 min. Fluorescent images were acquired using a confocal microscope (Leica SP8; Leica Microsystems, Wetzlar, Germany). All values were normalized against those of the control group. Measurements were assessed by an investigator blinded to the experimental conditions.

### Enzyme-linked immunosorbent assay (ELISA) and Seahorse assay

ELISA was performed to determine the activities of interleukin (IL)-1β, IL-6, and tumor necrosis factor (TNF)-α following the manufacturer’s instructions (Jiancheng, China). The Agilent Seahorse XF Cell Mito Stress Test was performed using an XF 96 Extracellular Flux Analyzer once HL-1 cells attained a confluence of 95% (5 × 10^3^ cells/well) on XF96 cell culture microplates (Agilent, Santa Clara, CA). The medium was replaced with an unbuffered XF medium (Agilent), and cells were equilibrated for 1 h at 37 °C without CO_2_ before the test. The XF96 Sensor Cartridges were prehydrated overnight in a calibration buffer before being loaded with compounds from the Seahorse XF Cell Mito Stress kit. Subsequently, integrated sensor cartridges and cell culture microplates were inserted into the XF96 Analyzer and subjected to the XF Cell Mito Stress Test protocol. Data were analyzed using the Seahorse Cell Mito Stress Test Report Generator, and statistical analysis was performed using GraphPad Prism 9 (La Jolla, CA).

### Western blot analysis

Heart tissues were processed for western blotting as described previously. Samples were disrupted using a ice-cold radioimmunoprecipitation assay buffer containing 10 mM Tris–Cl at pH 8.0, 1 mM ethylenediamine tetraacetic acid, 1% Triton X-100, sodium deoxycholate at 0.1%, and sodium dodecyl sulfate, also at 0.1%, enriched with 150 mM NaCl, 1 mM phenylmethylsulfonyl fluoride, and a mixture of protease inhibitors: aprotinin, leupeptin, and pepstatin at 0.02 mg/mL each. Following sonication, these lysates underwent clarification by centrifugation. Protein quantification was conducted utilizing the Bradford assay, and aliquots containing 50 to 100 mg of protein per lane were subjected to separation via sodium dodecyl sulfate–polyacrylamide gel electrophoresis. Postseparation, these proteins were electroblotted onto a polyvinylidene difluoride membrane and probed with specific antibodies, namely BCS1L (1:500, no. ab102808, Abcam) and FBXO7 (1:1000, no. ABN1038, Merck).

### Statistical analysis

All analyses were run in R (R version R 4.2.2 and R Studio version 1.0.143). Data are presented as mean ± standard deviation. The parametric Student’s *t*-test or the nonparametric Mann–Whitney test was used to compare between two groups. Parametric one-way analysis of variance (ANOVA) was performed for comparisons involving more than two groups, followed by the Bonferroni test for significance. A *p*-value < 0.05 was considered statistically significant.

## Results

### Identification of DEGs between healthy control individuals and patients with SCM

**Fig. 1:**
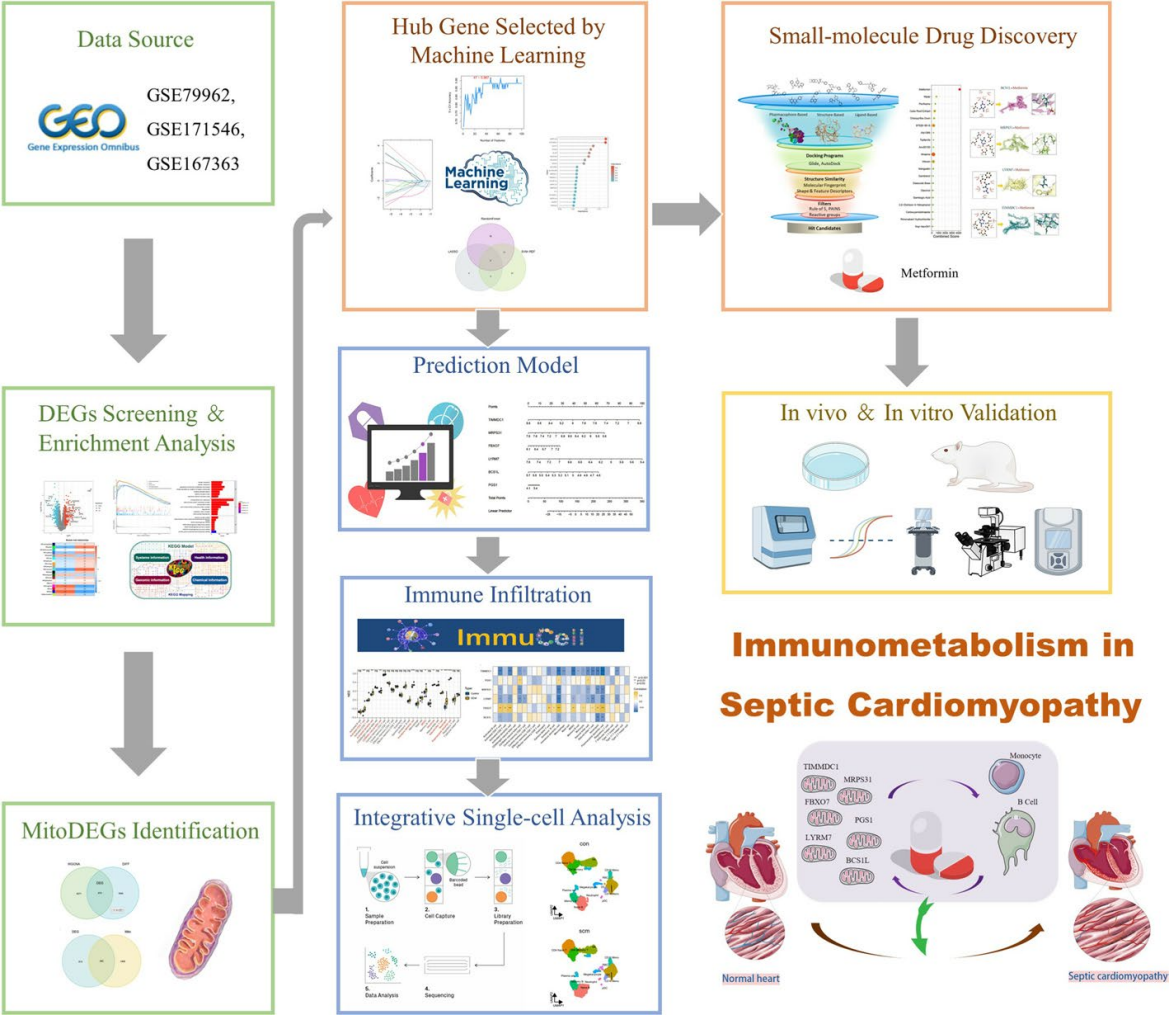
Flowchart of multistep analysis and validation strategy for bioinformatics data

**Fig. 2:**
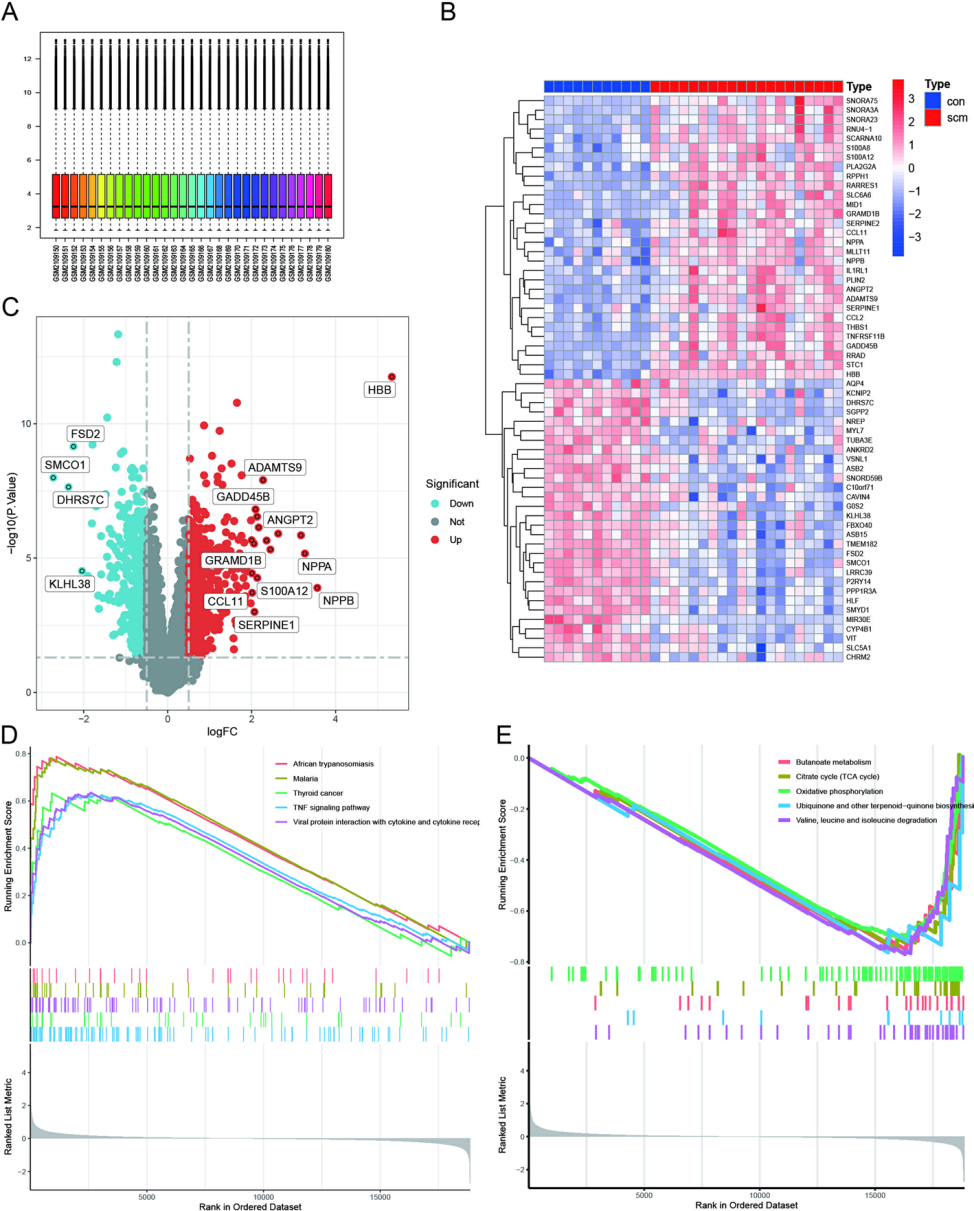
Data preprocessing for differentially expressed genes (DEGs). **A** Box plots of raw data normalized across samples. **B** Heatmap of DEG expression. **C** Volcano plot of DEG expression. **D**, **E** Gene set enrichment analysis (GSEA) identified the top five up- and downregulated pathways based on KEGG database

The flowchart of the study is presented in Fig. 1. By normalizing arrays from the GSE79962 dataset (Fig. 2A), we screened 2521 DEGs in myocardial samples from patients with SCM compared with nonfailing donors. The screening was based on the criteria of *P*-adjustment < 0.05 and log2 fold-change (FC) > 0.1. Specifically, 1126 and 1395 genes showed increased and decreased levels of expression, respectively (Additional file 2: Table S1). A volcano plot of these DEGs is shown, along with a heat map highlighting the top 30 upregulated and downregulated genes in Fig. 2B, C. The evaluation of pathway enrichment was performed by comparing the pathways between the SCM and control groups using GSEA. In the SCM group, the TNF signaling pathway, viral protein interaction with cytokine and cytokine receptors, thyroid cancer, and African trypanosomiasis were significantly enriched (Fig. 2D). Conversely, oxidative phosphorylation; citrate cycle (TCA cycle); butanoate metabolism; ubiquinone; other terpenoid-quinone biosynthesis pathways; and valine, leucine, and isoleucine degradation were significantly downregulated (Fig. 2E). These results suggest that mitochondrial dysfunction and immune dysregulation play a key role in SCM pathogenesis.

### Weighted gene coexpression network construction

**Fig. 3:**
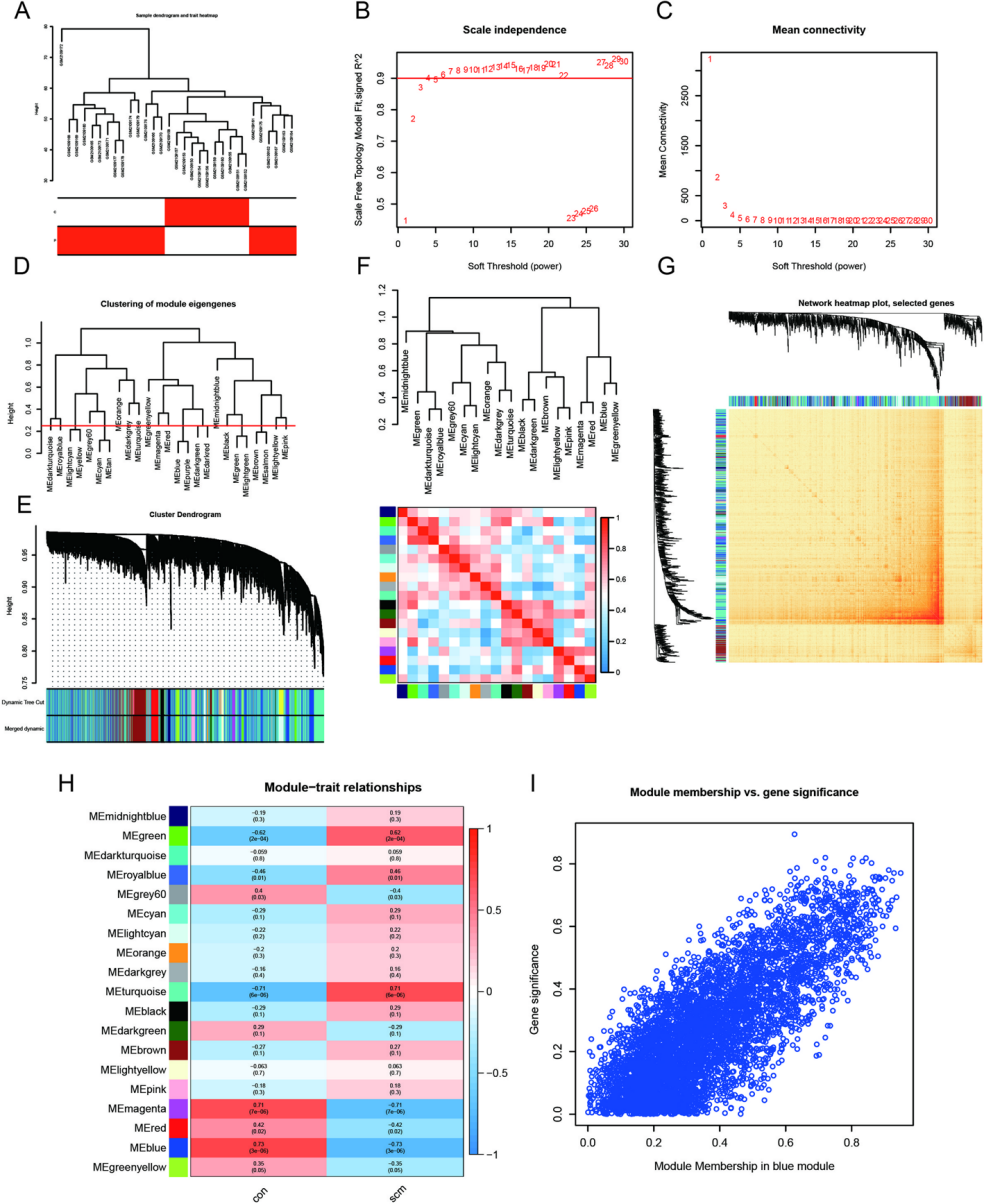
Enrichment levels in genomic weighted gene coexpression network analysis (WGCNA). **A** Sample clustering dendrogram with tree leaves representing each sample. **B**, **C** Soft thresholdβ = 7 and scale-free topological fit index (R^2^). **D** Similar modules were detected and combined by cutting clustered dendrograms at a height of 0.25. **E** Initial and merged modules within the clustering tree. **F** Collinear heat map of module feature genes. Red color represents a high correlation and blue color represents the opposite trend. **G** Clustering dendrogram of module feature genes. **H** Heat map of module-trait correlations. **I** scatter plot for the blue module

The GSE79962 dataset was obtained from the GEO data repository. A total of 11 normal samples and 20 SCM samples were selected for clustering after removing low-quality samples based on a predetermined threshold, as shown in Fig. 3A. Subsequently, a soft threshold of seven was used when R^2^ > 0.9 and the average connectivity was high, as shown in Fig. 3B, C. By merging the strongly associated modules with a clustering height threshold of 0.25 (Fig. 3D), 19 modules were identified for further analysis. The primed and merged modules are presented as clustering trees in Fig. 3E. The subsequent analysis involved examining the correlation between modules, which confirmed a lack of significant connection between them (Fig. 3F). To demonstrate the reliability of module delineation, transcriptional correlation within modules was assessed. No substantial linkage between modules was found (Fig. 3G). Furthermore, the relationship between modules and clinical symptoms was investigated by utilizing frontal correlations between ME values and clinical features. The blue module exhibited the highest correlation with both normal and SCM groups (*P* = 3e−08) (Fig. 3H). Significant modules with clinical relevance were identified. The strongest association was found between the blue module and clinical features in the module membership (MM) versus gene significance (GS) scatter plot (Fig. 3I). All genes within the blue modules were selected for further analysis (Additional file 3: Table S2).

### Identification and functional analysis of MitoDEGs in the context of SCM

After conducting an analysis using the WGCNA method to identify critical module genes and the limma method to identify DEGs, a Venn diagram was drawn and 875 overlapping genes were obtained (Additional file 1: Fig. S1A). Mitochondria-related genes were retrieved from the MitoCarta3.0 database. The mitochondria-related genes intersected with the above 875 overlapping genes were identified as MitoDEGs. In total, we identified 262 MitoDEGs (Additional file 1: Fig. S1B). Subsequently, functional analysis was conducted to gain further insight into the biological functions of these MitoDEGs. As evident from the results of DO analysis, these MitoDEGs were found to be associated with cardiomyopathy, muscle tissue disease, Parkinson’s disease, and mitochondrial metabolism disease (Additional file 1: Fig. S1C). GO enrichment analysis revealed that the MitoDEGs were involved in the regulation of cellular respiration, oxidative phosphorylation, the electron transport chain, NADH dehydrogenase (ubiquinone) activity, and electron-transfer activity (Additional file 1: Fig. S1D). KEGG analysis showed their association with diabetic cardiomyopathy, ROS, carbon metabolism, and TCA cycle (Additional file 1: Fig. S1E, F).

### Selection of candidate hub genes using machine learning algorithms

**Fig. 4:**
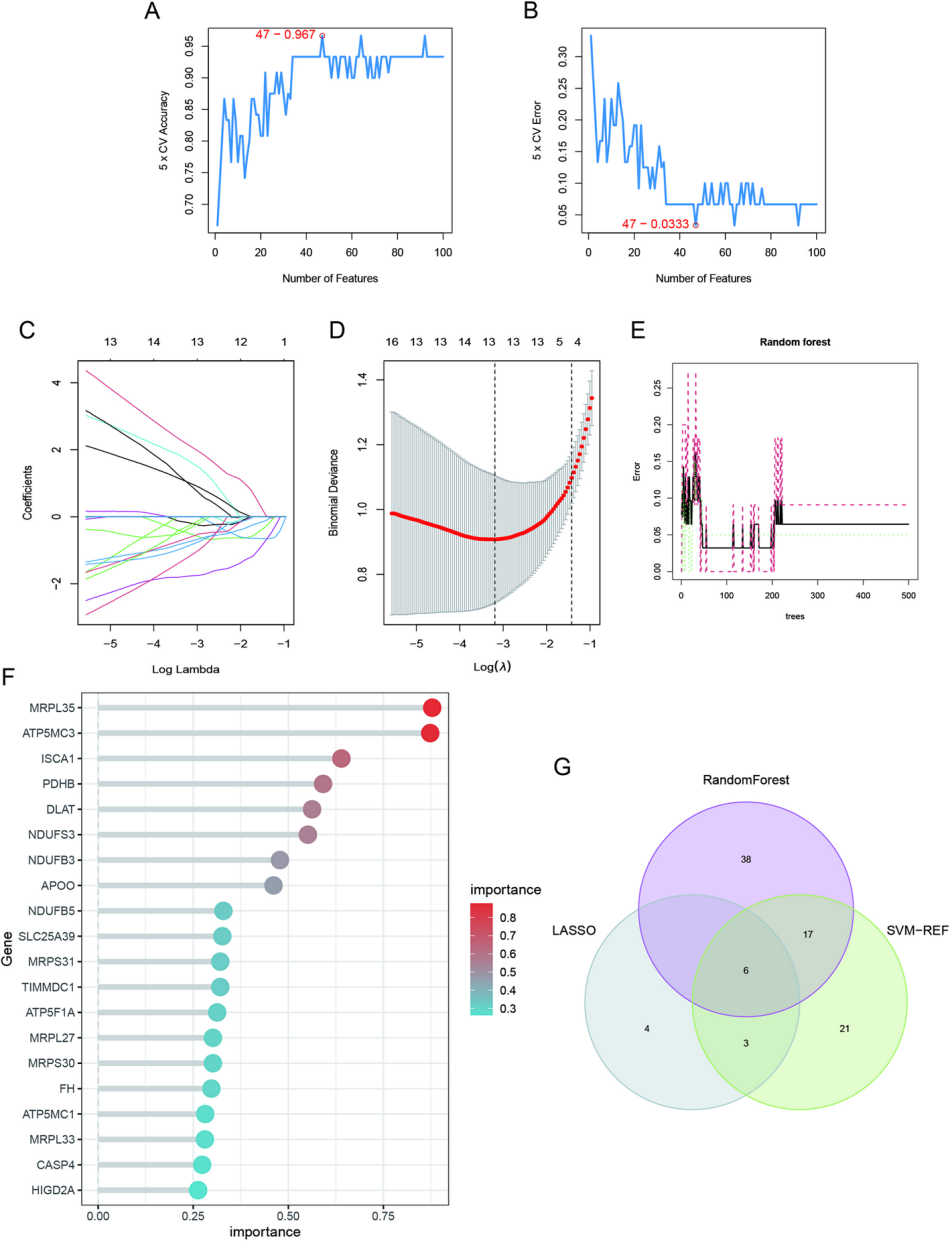
Feature gene selection. **A**, **B** Signature gene expression was screened based on the support vector machine recursive feature elimination (SVM-RFE) algorithm. **C**, **D** Adjusting feature selection using the least absolute shrinkage and selection operator (LASSO) algorithm. **E** Random forest error rate versus the number of classified trees. **F** The top 20 key genes. **G** Venn diagram of the six hub genes obtained from the intersection of results from SVM-RFE, RF, and LASSO algorithms

Three machine-learning algorithms were used to screen feature genes among the set of 262 MitoDEGs. Specifically, SVM-RFE identified 47 genes with the highest accuracy of 0.967 and the lowest error of 0.033 (Additional file 4: Table S3) (Figs. 4A, B); LASSO regression analysis predicted 13 genes among the statistically significant univariate variables (Fig. 4C, D) (Additional file 5: Table S4); random forest and feature selection were employed to determine the relationship between error rate, classification tree numbers, and 61 genes with relative importance (Fig. 4E–G) (Additional file 6: Table S5). To obtain a robust gene signature for SCM, genes that overlapped among the three aforementioned methods were obtained using a Venn diagram. Six hub genes, namely BCS1L, FBXO7, LYRM7, MRPS31, PGS1, and TIMMDC1, were obtained as shown in Additional file 1: Fig. S2A. Results of correlational analysis among the six hub genes are shown in Additional file 1: Fig. S2A. BCS1L, LYRM7, MRPS31, and TIMMDC1 decreased significantly in the SCM group, whereas FBXO7 and PGS1 increased markedly in SCM samples compared with controls, as shown in Additional file 1: Fig. S2B–G.

### Modeling and testing a diagnostic nomogram model for SCM

**Fig. 5:**
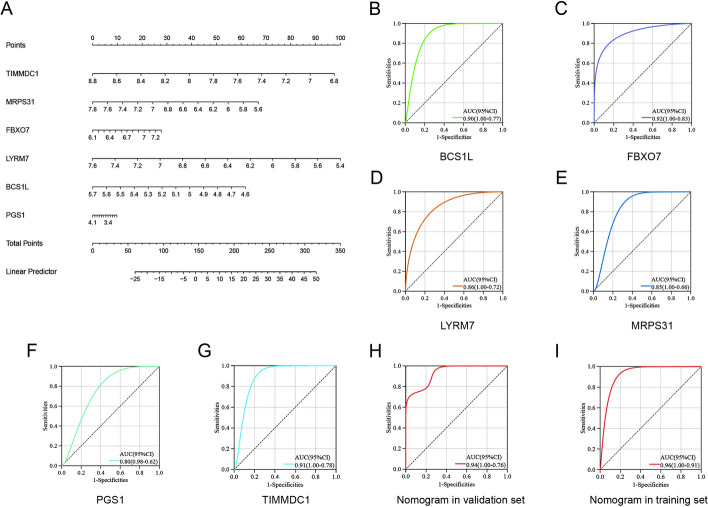
Nomogram construction and evaluating the diagnostic value. **A** Visualization of the nomogram for SCM diagnosis. **B**–**I** ROC curve for each candidate gene (BCS1L, FBXO7, LYRM7, MRPS31, PGS1, and TIMMDC1) and the nomogram in both the training and validation sets show its significant diagnostic value for SCM

A nomogram based on the six candidate hub genes was constructed (Fig. 5A). The diagnostic specificity and sensitivity of each gene, and the nomogram itself, were evaluated using the ROC curve. The AUC and its corresponding 95% CI were calculated for each gene. The results were as follows: BCS1L (AUC 0.90, CI 0.77–1.00), FBXO7 (AUC 0.92, CI 0.83–1.00), LYRM7 (AUC 0.86, CI 0.72–1.00), MRPS31 (AUC 0.85, CI 0.66–1.00), PGS1 (AUC 0.80, CI 0.62–0.98), and TIMMDC1 (AUC 0.91, CI 0.78–1.00) (Fig. 5B–G). The AUC of the nomogram model was 0.96 (95% CI: 0.91–1.00) in the training set and 0.94 (95% CI, 0.76–1.00) in the GSE171546 validation set. These findings, therefore, suggest that all candidate genes demonstrated have a substantial diagnostic value for SCM, while the constructed nomogram demonstrated the highest diagnostic efficacy.

### Gene set enrichment of the hub genes

The potential biological function of the six hub genes was investigated in greater detail. GSEA was performed to query the Hallmark Pathways in mSigDB. GSEA was performed between high- and low-expression groups of BCS1L, FBXO7, LYRM7, MRPS31, PGS1, and TIMMDC1 in patients with SCM. As shown in Additional file 1: Fig. S3A–D, genes with low expression of BCS1L, LYRM7, MRPS31, and TIMMDC1 were significantly enriched in inflammatory response, interferon-gamma response, interferon alpha response, TNFα signaling via NFκB, hypoxia, and IL6-JAK-STAT3 signaling pathways. As shown in Additional file 1: Fig. S3E, F, the group with high expressions of FBXO7 and PGS1 were also significantly enriched in allograft rejection, inflammatory response, interferon alpha response, TNFα signaling via NFκB, hypoxia, IL6-JAK-STAT3 signaling, and unfolded protein response cascades. Biological differences between patients with SCM and the healthy control group were further investigated using ssGSEA. Correspondingly, the correlation between signature gene expression and ssGSEA scores of hallmark gene sets was estimated using the “corrplot” package (Additional file 1: Fig. S3G). Signature genes showed significant and strong correlations with the following hallmark gene sets including oxidative phosphorylation, fatty acid metabolism, bile acid metabolism, inflammatory response, IL6-JAK-STAT3 signaling, IL2-STAT5 signaling, TGF-β signaling, and TNFα signaling via NFκB.

### Immune cell infiltration in SCM

The ssGSEA algorithm was used to analyze the infiltration of 28 immune cell types, and the SCM and control groups in the GSE79962 dataset were compared with investigate variations in the immune landscape. Significant disparities in the myocardial infiltration of activated CD4 T cell, activated dendritic cell, CD56dim natural killer cell, immature dendritic cell, MDSC, monocyte, natural killer T cell, neutrophil, and plasmacytoid dendritic cell between the DCM and CON groups (*P* < 0.05). All the aforementioned immune cell types were significantly enriched in the microenvironment of patients with SCM (Additional file 1: Fig. S3A, B). As shown in Additional file 1: Fig. S4C, additional analysis of the infiltrating immune cells in SCM revealed several significant correlations between these cells. The strength of these correlations was further quantified using scores. Notably, the strongest synergistic effect was observed between memory B cells and macrophages (0.85), followed by macrophages and eosinophils (0.82), monocytes and memory B cells (0.82), immature dendritic cells and gamma delta T cells (0.81), and monocytes and macrophages (0.80).

### Relationship between hub MitoDEGs and immune cells

**Fig. 6:**
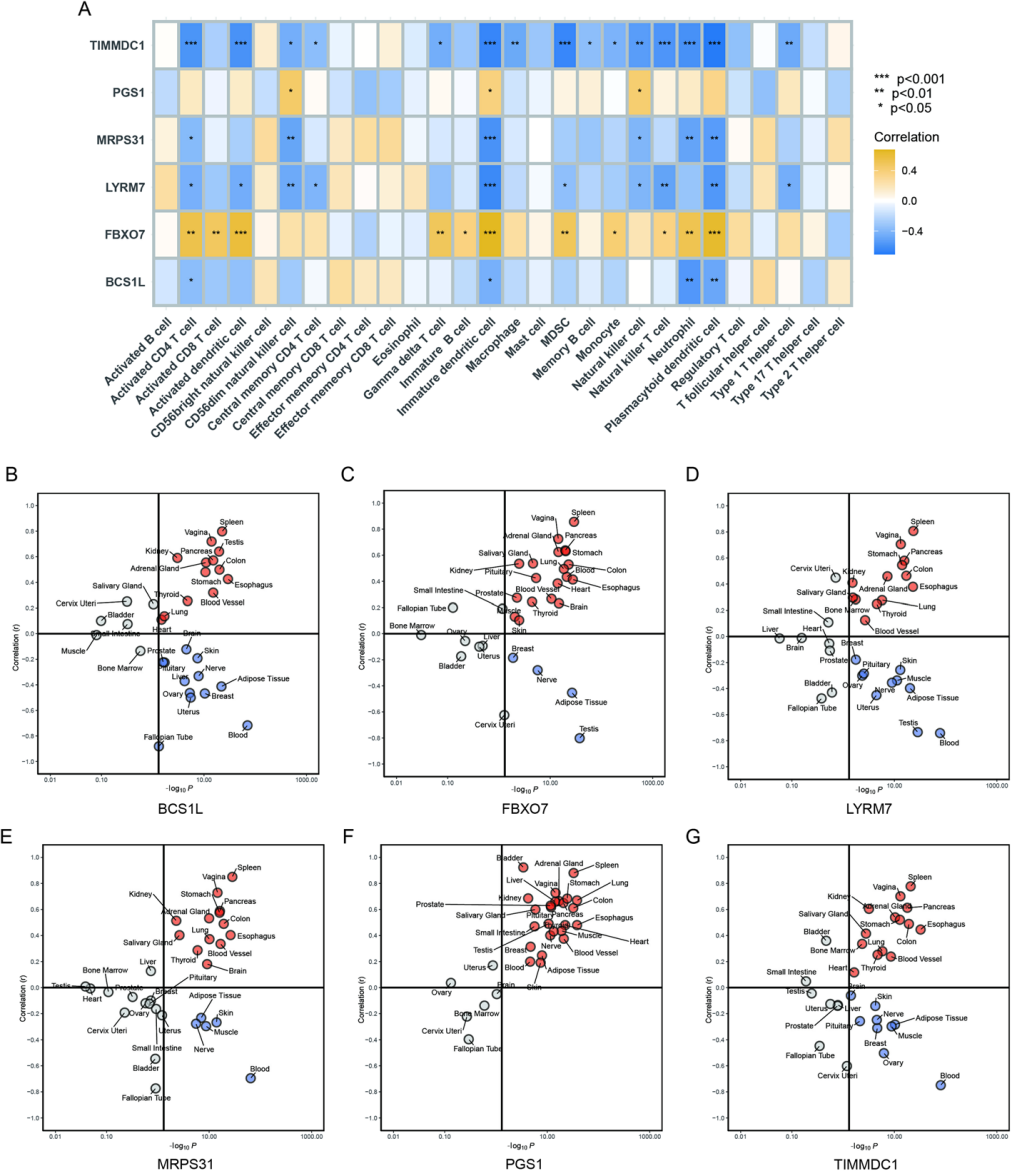
Correlation and pantissue analysis between six hub genes and inflammation disorder. **A** Correlation between hub genes and immune cells. **B**–**G** Pantissue analysis of the correlation between the expression of six hub genes and hallmark-inflammatory responses in 31 types of tissues based on the GTEx database

We assessed the potential relationship between the six hub MitoDEGs and immune cells. Correlational analyses revealed a generally positive relationship between the upregulated hub genes of SCM (FBXO7 and PGS1) and the upregulated infiltration of immune cells but a generally negative relationship between the downregulated hub genes (BCS1L, LYRM7, MRPS31, and TIMMDC1) and the infiltration of immune cells (Fig. 6A). The most significantly positive correlation was found between FBXO7 expressions and immature dendritic cell infiltrations (*r* = 0.67, *P* < 4.78e^−5^). The negative correlation between TIMMDC1 expressions and plasmacytoid dendritic cell infiltration was most significant (*r* = − 0.70, *P* < 1.39e^−5^).

Meanwhile, to further explore the MitoDEGs-inflammation association during SCM, we plotted the correlation between ssGSEA scores of hallmark-inflammatory response and hub gene expression in pantissue clinical samples through the Genotype-Tissue Expression (GTEx) database (https://gtexportal.org/home/). Six hub genes were correlated significantly with the inflammatory response in most tissues (Fig. 6B–G).

### Single-cell RNA-seq analysis

**Fig. 7:**
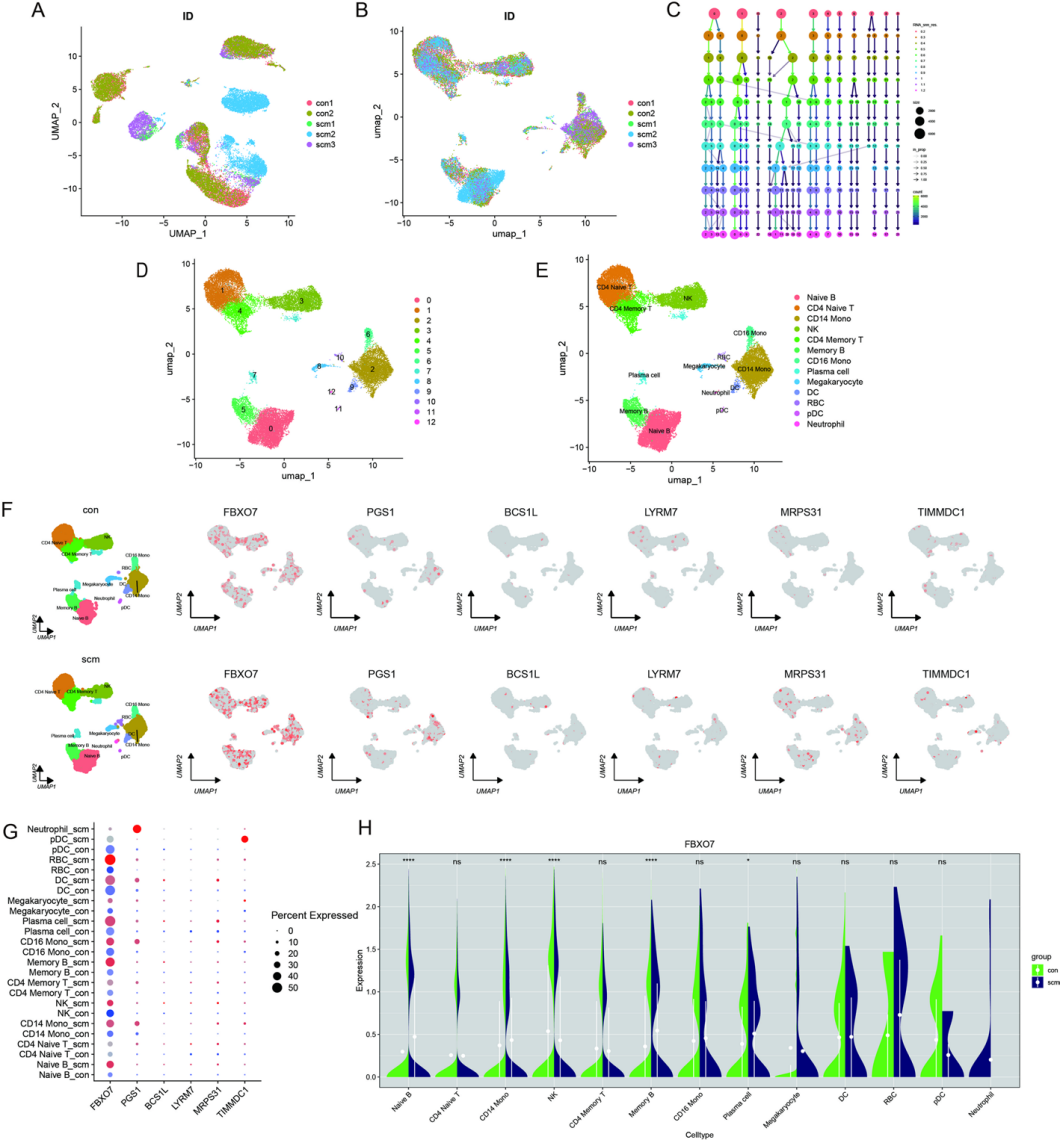
Comparison of single-cell analysis before and after normalization. **A** UMAP plots representing batch effects from replicates before Harmony, **B** UMAP plots showing the correction of batch effects after Harmony. **C** Clustree plot for determining resolution with principal components (PCs). **D** Unified manifold approximation and projection clustering into 13 clusters. **E** Cells from human peripheral blood samples were annotated using CellMarker and singleR. **F** FeaturePlots showing the expression pattern of FBXO7, PGS1, BCS1L, LYRM7, MRPS31, and TIMMDC1 in peripheral blood mononuclear cells (PBMCs) from SCM and control groups. **G** Dot plot shows the expression levels of hub genes in each cell cluster. **H** The violin plot displays the gene expression of FBXO7 in each cell cluster. *p < 0.05, **p < 0.01, ***p < 0.001

Single-cell RNA-seq data of PBMCs from two healthy participants and three patients with sepsis were obtained from GSE167363. Cells with gene detection counts per cell exceeding 2500 or falling below 200, as well as cells with mitochondrial percentages exceeding 5%, were excluded to ensure data quality. The gene expression matrices were then subjected to batch effect removal via Harmony (Fig. 7A, B). To determine the optimal resolution for unsupervised clustering, performances of 11 different resolution values were compared using the clustree package (Fig. 7C). A resolution of 0.3 was chosen to accurately differentiate cell types based on their preassigned annotations. UMAP revealed the presence of 13 distinct cell clusters, each labeled with a distinct color (Fig. 7D). Considering the expression patterns of marker genes, the clustering results obtained through UMAP were further refined and annotated using singleR and CellMarker (Fig. 7E). The expression pattern of six hub genes was depicted in the PBMS UMAP plots of healthy participants and patients with SCM. FBXO7 was significantly upregulated in the SCM group (Fig. 7F). Dotplot analysis indicated that FBXO7 expression in the control and SCM groups was variable among different cell clusters (Fig. 7G). Quantitative analysis of FBXO7 expression, as shown in the violin plot, revealed an increase in FBXO7 expression in the naive B cell, CD14^+^ monocyte, memory B cell, and plasma cell (Fig. 7H).

### Overview of the mitochondria/inflammation-related pathway of interest at single-cell resolution

**Fig. 8:**
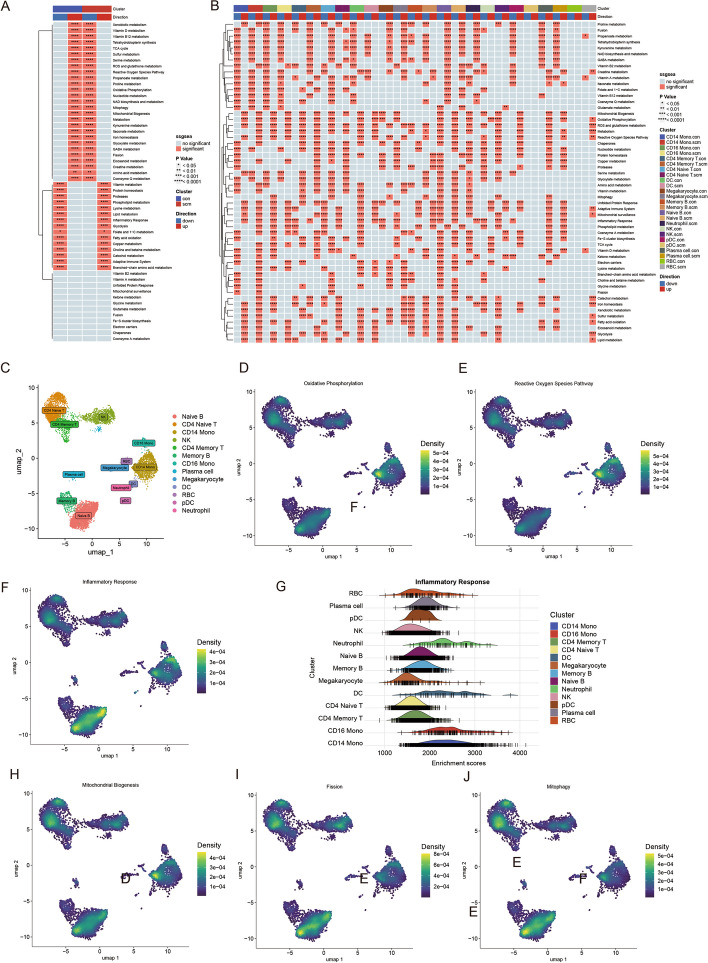
Overview of the mitochondria/inflammation-related pathways of interest at single-cell resolution. **A**, **B** Heatmap of gene set enrichment analysis (GSEA) scores for mitochondria/inflammation-related pathways among different patient groups and cell subsets. **C** UMAP of SCM scRNA-seq datasets with cluster annotations. **D**–**J** Density scatterplot and ridge plot reflecting the distribution of mitochondria/inflammation-related pathways of interest in the SCM group. *p < 0.05; **p < 0.01; ***; *p* < 0.001

Our previous study demonstrated that disorders of the immune system and mitochondrial metabolism system play significant roles in the pathogenesis and pathophysiology of SCM. Consequently, we utilized the “irGSEA” package to investigate distributions of the aforementioned pathways using scRNA-seq gene set enrichment analysis. Most mitochondrial metabolism-related pathways were downregulated in the SCM group. In contrast, inflammation-related pathways were upregulated in the SCM group (Fig. 8A). The heatmap of GSEA scores for cell subsets in the control and SCM groups showed varied distribution of the mitochondria/inflammation-related pathways across subgroups (Fig. 8B). The density scatterplot showed the distribution of the OXPHOS, ROS, mitochondrial biogenesis, mitophagy, mitochondrial fission, and inflammatory response pathways in the SCM group (Fig. 8C–J). The ridge plot demonstrated that the inflammatory response pathway was predominantly enriched in neutrophil, DC, and monocyte subgroups of patients with SCM. This observation carries significant implications since these cell subtypes are well-known for their essential roles in the immune system. The finding suggests that activation of inflammation and mitochondrial dysfunction in these particular cell subtypes can cooperate and may even function synergistically in the pathogenesis of SCM.

### Prediction of candidate drugs

**Fig. 9:**
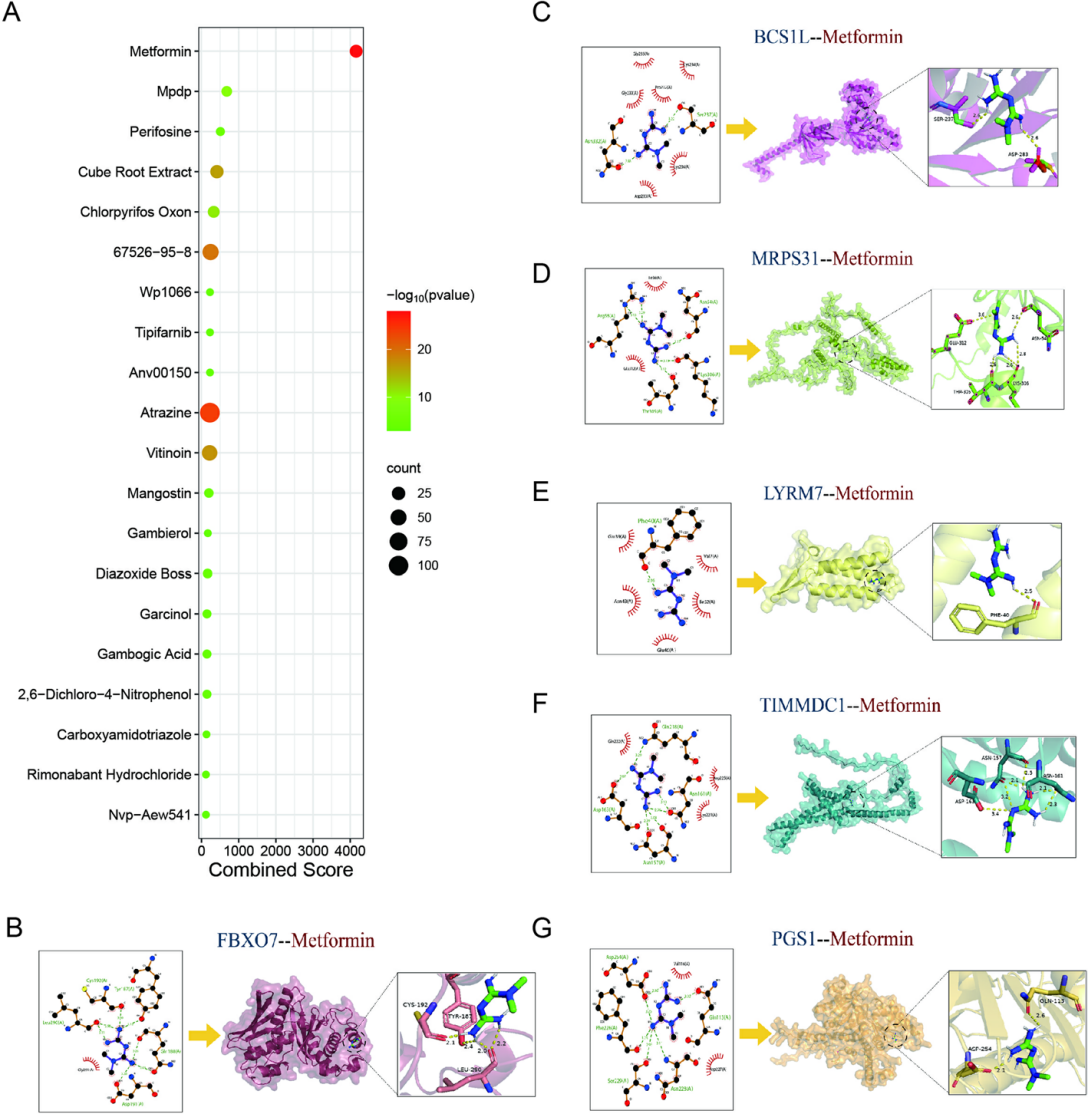
Prediction of the top 20 candidate drugs for SCM based on mitoDEGs. **A** The top 20 most significant candidate compounds were predicted for MitoDEGs using the DSigDB database. **B**–**G** Molecular docking between MitoDEGs and metformin

Using the DSigDB database from the Enrichr website, we searched for potentially effective interventional drugs targeting the hub genes. Figure 9A shows the top 20 potential chemical compounds based on their combined score and adjusted *p*-value. Metformin, with the highest combined score, was the most promising drug molecule for the treatment of SCM. We further investigated how metformin exerted its protective effects on mitochondria in SCM. The binding between metformin and six key targets (Fig. 9B–G) was investigated using molecular docking. The molecular docking validation confirmed that the relative binding energies of metformin and FBXO7/BCS1L/PGS1/LYRM7/TIMMDC1/MRPS31 fall within the range suitable for their interaction (Table 1). FBXO7 was found to possess the highest binding energies with metformin.

**Table 1:** Binding energy of Metformin to six hub targets

Target	FBXO7	BCS1L	PGS1	LYRM7	TIMMDC1	MRPS31
Binding energy (kcal/mol)	−5.6	−5.4	−5.0	−4.7	−4.4	−4.1

### Experimental validation

**Fig. 10:**
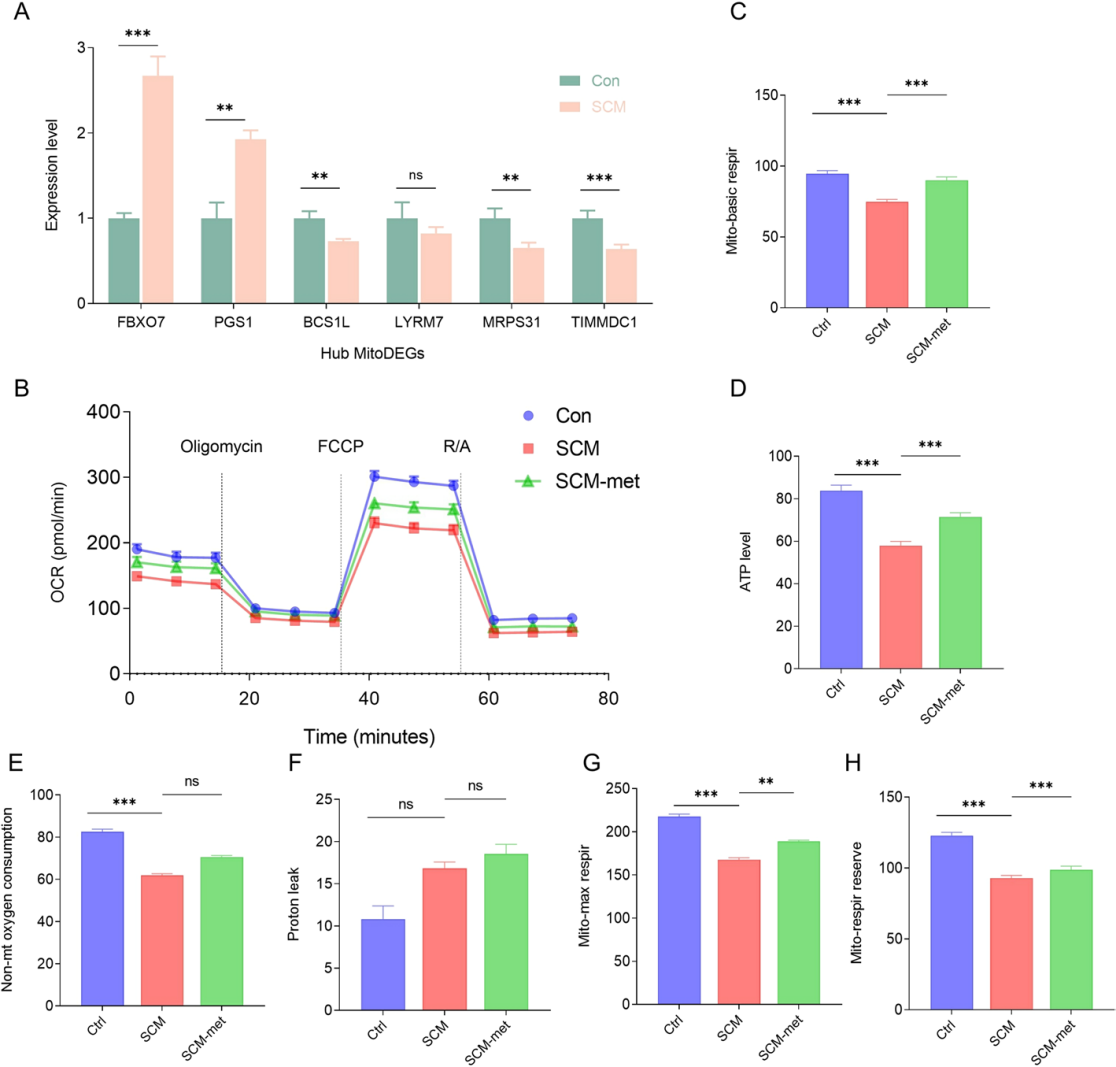
Confirmation of hub MitoDEG expression and the key role of mitochondrial dysfunction in the pathogenesis of SCM. **A** mRNA expression of the hub MitoDEGs between the control and SCM groups. **B** Analysis of HL-1 mitochondrial metabolism with Seahorse XFe96 Analyzer. OCR was monitored continuously at baseline and after the addition of oligomycin (2 mM), FCCP (1 mM), and R/A (0.5 mM). **C**–**H** Basal respiration, maximal respiration, nonmitochondrial oxygen consumption, spare respiratory capacity, proton leak, and ATP production levels. Mitochondrial metabolism is impaired in the SCM group. OCR, oxygen consumption rate; FCCP, carbonyl cyanide-4-phenylhydrazone; R/A, rotenone and antimycin A (*N* = 8 independent cell samples per group). *p < 0.05, **p < 0.01, ***p < 0.001

**Fig. 11:**
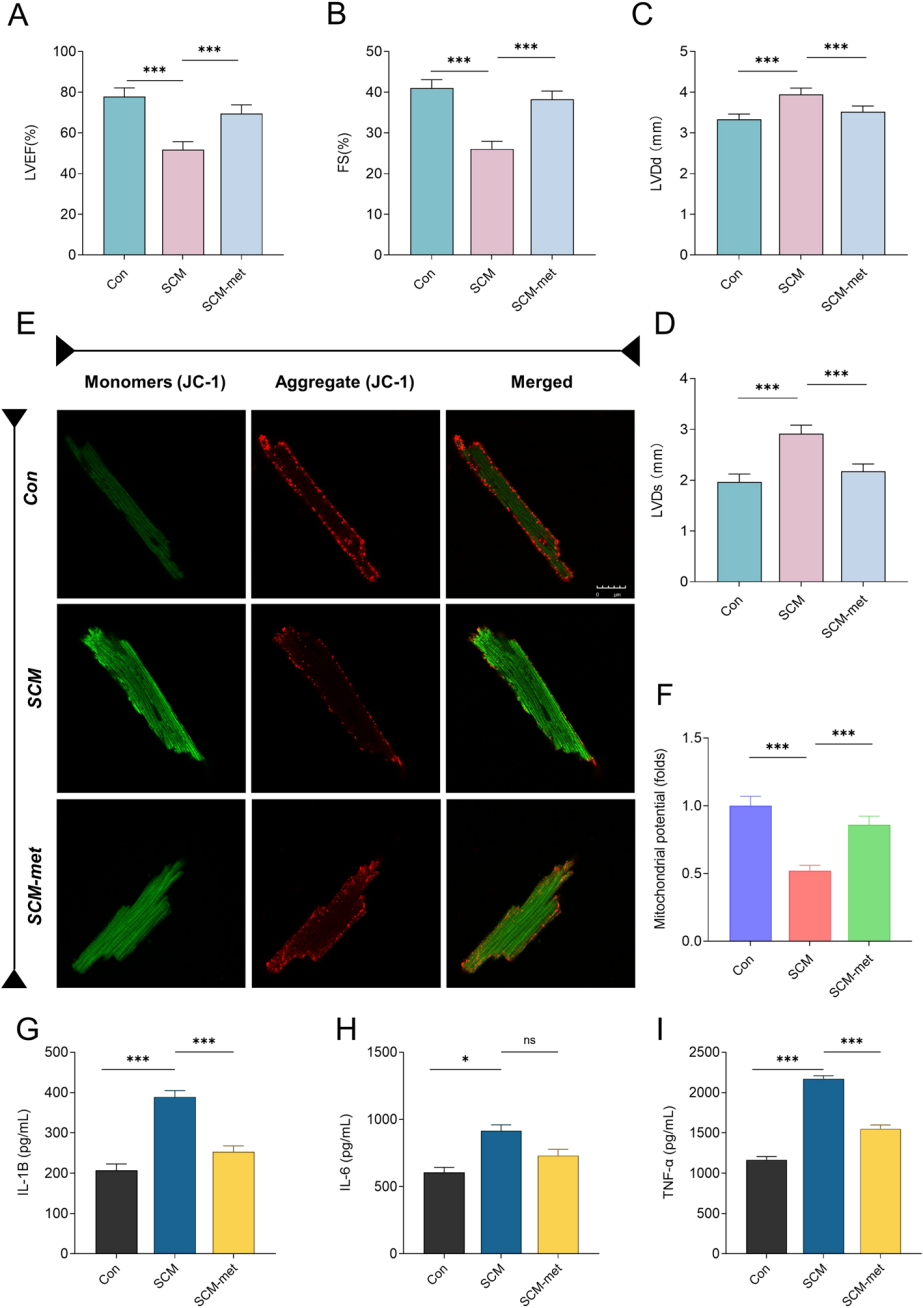
Metformin improves cardiac function by alleviating mitochondrial injury and inflammatory response in mice with SCM. **A**–**D** Results of echocardiography. LVEF, left ventricular ejection fraction; FS, left ventricular fraction shortening; LVDd, left ventricular diastolic dimension; LVDs, left ventricular systolic dimension. **E**, **F** Analysis of the mitochondrial membrane potential in isolated cardiomyocytes loaded with JC-1. **G**–**I** Comparison of expression of inflammation factors between different groups (*N* = 6 mice or eight independent cell samples per group). *p < 0.05, **p < 0.01, ***p < 0.001

Using quantitative reverse-transcription polymerase chain reaction (qRT-PCR; *n =* 8 in each group), the expression of six hub MitoDEGs (FBXO7, PGS1, BCS1L, LYRM7, MRPS31, and TIMMDC1) in HL-1 cells treated with LPS was confirmed. FBXO7 and PGS1 were significantly increased in the SCM group compared with the control group (*P* < 0.05), whereas BCS1L, MRPS31, and TIMMDC1 were significantly decreased in the SCM group (*P* < 0.05) (Fig. 10A). Given the central role of mitochondrial metabolism in the SCM, we investigate whether LPS treatment increased the susceptibility of the myocardium to sepsis by altering mitochondrial respiration function using the Seahorse XF Cell Mito Stress Test (*n* = 8 in each group). Upon LPS treatment, basal, reserve, maximal respiratory capacity, and ATP levels were all relatively impaired in the SCM group, and metformin treatment effectively mitigated the impairment in mitochondrial metabolism (Fig. 10B–H). We validated our results by investigating these associations in animal models. Compared with the control group, echocardiography revealed significantly decreased EF% and FS% in the SCM group (*P* < 0.05), while LVDd and LVDs were significantly increased (*P* < 0.05; *n =* 6 in each group) (Fig. 11A–D). Metformin intervention in the SCM model significantly reversed the aforementioned changes. To examine the hypothesis that the cardioprotective effects of metformin on SCM are mediated by mitochondria, primary cardiomyocytes were stained with JC-1 dye and imaged by confocal microscopy. JC-1 fluorescence analysis revealed decreased mitochondrial membrane potential in the SCM group, which was restored following metformin treatment (*n =* 8 in each group, Fig. 11E, F). ELISA was performed to assess whether metformin affected inflammation by observing the changes in the levels of inflammatory factors (*n =* 6 in each group). Metformin intervention significantly reversed the SCM-induced increase in TNF-α/IL-1β levels (Fig. 11G–I).

**Fig. 12:**
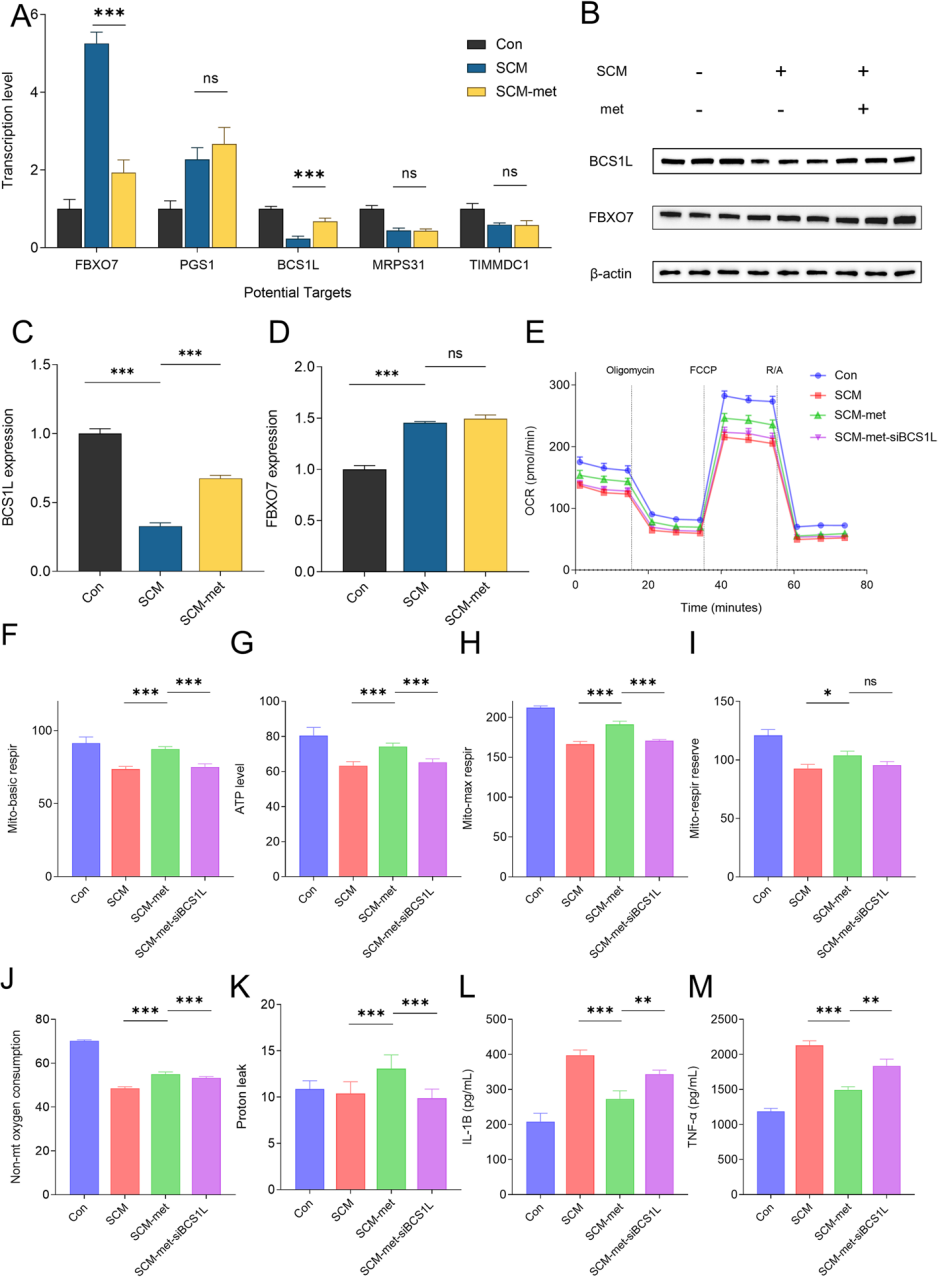
Metformin can potentially regulate the immune-metabolic microenvironment in SCM by targeting BCS1L. **A** mRNA expression of potential targets in the control, SCM, and SCM–metformin groups. **B**–**D** Proteins were isolated from cardiomyocytes in the control, SCM, and SCM–metformin groups, and the levels of BCS1L and FBXO7 were determined by western blotting. **E**–**K** Analysis of HL-1 mitochondrial metabolism with Seahorse XFe96 Analyzer. OCR was monitored continuously at baseline and after adding oligomycin (2 mM), FCCP (1 mM), and R/A (0.5 mM) to compare basal respiration, maximal respiration, nonmitochondrial oxygen consumption, spare respiratory capacity, proton leak, and ATP production levels among the control, SCM, SCM–metformin, and SCM–metformin–siBCS1L groups. **G**–**I** Comparison of levels of inflammation factors among different groups (*N* = 6 mice or eight independent cell samples per group). *p < 0.05, **p < 0.01, ***p < 0.001

To identify the therapeutic targets of metformin intervention in cardiomyocytes affected by SCM, we examined the expression of five potential biomarkers (FBXO7, PGS1, BCS1L, MRPS31, and TIMMDC1) by qPCR in the control, SCM, and SCM + metformin groups (*n =* 6 in each group, Fig. 12A). Metformin intervention could reduce the upregulated transcriptional levels of FBXO7 in the SCM group and restore the downregulated transcriptional levels of BCS1L. Western blot analysis confirmed that the interventional effect of metformin on BCS1L target was more robust (Fig. 12B–D). We further constructed a BCS1L-knockdown cell model using siRNA to validate the interventional target. Seahorse XF Cell Mito Stress Test analysis demonstrated that metformin intervention significantly ameliorated mitochondrial dysfunction in cardiomyocytes following SCM stimulation. However, the cardioprotective effects of metformin on mitochondria were attenuated in the BCS1L-knockdown condition (*n =* 8 in each group, Fig. 12E–K). The levels of inflammatory factors under various conditions were observed using ELISA. Compared with the SCM + metformin group, a significant increase in the expression of inflammatory factors (IL-1β/TNFα) was found in the SCM + metformin + si-BCS1L group (*n =* 8 in each group, Fig. 12L, M). These findings suggest that BCS1L may be a key target of metformin in modulating the cardiac immune-metabolic microenvironment in SCM.

## Discussion

Severe effects of sepsis impose a substantial burden on the health care system owing to the high morbidity and mortality rates. SCM is among the leading causes of mortality in patients with sepsis [6, 35]. Unfortunately, a lack of understanding of the mechanism and consensus on diagnostic criteria impedes early diagnosis and effective treatment of SCM. Therefore, there is an urgent need to enhance the understanding of SCM pathogenesis and identify promising drugs and novel therapeutic targets for its treatment.

By integrating bioinformatics methods, we identified DEGs from SCM-related human microarray datasets from the GEO repository. It was observed that the DEGs were enriched in mitochondria/immune-associated pathways such as oxidative phosphorylation, the TCA cycle, and the TNF signaling pathway. Because the proper functioning of the cardiac contractile and relaxation mechanisms is fundamentally dependent on the sustained energy supply, mitochondrial dysfunction, and metabolic disorders are closely associated with various cardiovascular diseases [18, 36–38]. Sepsis is characterized by an aberrant host immune response to infection. Among the host determinants, the innate immune system is vital for prompt recognition and elimination of pathogens. Nevertheless, in sepsis, the innate immune response may be hyperactive or hypoactive, resulting in a cytokine storm or immunosuppression, respectively [39, 40]. In addition, the immune dysregulation can trigger a cascade of events including impairment in cellular metabolism [17, 41, 42]. Based on these findings, the purpose of the study was to further investigate the regulatory role of mitochondrial metabolism and immune dysregulation in the onset and progression of SCM, along with seeking potential therapeutic targets and agents for SCM.

To our knowledge, mitochondria-related DEGs involved in courses of SCM have not so far been screened in related bioinformatics studies. Our research utilized MitoCarta 3.0, a comprehensive database of mitochondrial genes and associated biological processes, to acquire SCM-related genes associated with mitochondria [43]. A total of 262 MitoDEGs were chosen for further analysis. Previously, machine learning algorithms such as SVM and RF have been utilized successfully to identify latent patterns and construct prediction models based on the most accurate determinants in training datasets [44, 45]. Herein, we used three machine algorithms (SVM–RFE, LASSO, and RF) and identified six hub mitoDEGs.

Mitochondrial dysfunction is a key etiological factor in the pathology of SCM. TIMMDC1, MRPS31, FBXO7, PGS1, LYRM7, and BCS1L are key regulatory genes for mitochondrial metabolism. TIMMDC (translocase of inner mitochondrial membrane domain-containing) is a member of the inner mitochondrial membrane translocase family. The TIMMDC1 gene encodes protein M5-14, which is essential for the assembly of mitochondrial NADH and the formation of the complex I membrane arm. Previous studies indicate that the depletion of TIMMDC1 noticeably inhibits the growth and migration of 95D cells by reducing mitochondrial viability, membrane potential, and ATPase activity [46]. Kang et al. identified TIMMDC1 as the key regulator in the pathogenesis of SCM, consistent with our findings [47]. The mitochondrial ribosomal protein S31 (MRPS31) is a small subunit protein necessary for the assembly of the mitochondrial ribosome. It also plays a critical role in the translation of mitochondrial DNA into functional proteins, thus constituting key modules of OXPHOS. Intriguingly, the downregulation of MRPS31 is associated with reduced cellular bioenergetics. In hepatoma cell lines such as JHH5 and HepG2, MRPS31 suppression has been shown to enhance the invasiveness of hepatoma cells and disrupt the entire assembly of mitoribosomes [48].

FBXO7 is a component of the SKP1–Cullin1–F-box (SCF) SCFFBXO7 E3 ligase complex, responsible for identifying particular substrates [49]. FBXO7 plays a crucial role in the regulation of important mitochondrial events such as mitophagy and the proteasome process of protein quality control [50, 51]. Inhibiting FBXO7 effectively suppresses inflammation by disrupting the interaction between mitochondrial kinase, PINK1, and the ubiquitin machinery, thus improving mitochondrial quality [52]. However, additional research is necessary to determine the precise prognostic significance of FBXO7 in patients with SCM. PGS1 is crucial in the biosynthesis of cardiolipin, which is important in mitochondrial biogenesis as it directly binds to mitochondrial proteins and stabilizes multiprotein mitochondrial complexes. Wai et al. demonstrated that downregulation of PGS1 decreases cardiolipin levels in mitochondria and rescues mitochondrial dynamics in OPA1-mutant fibroblasts by inhibiting mitochondrial fission [53].

LYRM7 is a newly identified gene involved in the final stages of assembling mitochondrial complex III. It encodes nuclear-encoded mitochondrial matrix protein which stabilizes UQCRFS1 and conveys it as a chaperone to the CIII complex. Defects in LYRM7 are implicated in mitochondrial complex III (cIII) deficiency. Ferrero et al. discovered that a homozygous mutation in LYRM7/MZM1L is correlated with early onset encephalopathy and lactic acidosis, which may be attributed to severe suppression of OXPHOS [54]. BCS1L encodes a homolog of the *Saccharomyces cerevisiae* bcs1 protein, which is important for the assembly of complex III. A recent study has demonstrated that cIII‐deficient Bcs1l ^p.S78G^ mice are at a high risk of lethal mitochondrial cardiomyopathy, which is accompanied by ROS overproduction, a decrease in mitochondrial respiration, and damage to mitochondrial ultrastructure [55, 56].

To facilitate the application of the theoretical diagnostic genome in routine clinical settings, a multivariable nomogram involving all six hub MitoDEGs was constructed. The expression of each gene was quantified and converted to a score, which was then correlated with a linear predictor. An abnormal increase in the linear predictor of patients with sepsis can aid in early monitoring and treatment, making the nomogram a valuable diagnostic tool for SCM.

To further demonstrate the action of hub MitoDEGs in SCM, GSEA was performed. Our findings indicated significant enrichment of inflammation-related pathways in the high-expression subgroup of FBXO7/PGS1 and the low-expression subgroup of BCS1L/LYRM7/MRPS31/TIMMDC1, including inflammatory response, interferon alpha response, TNFα signaling via NFκB, and IL6-JAK-STAT3 signaling pathways. Considering that mitochondrial dysfunction and immune disorder frequently interact and affect each other, the ssGSEA algorithm was employed to quantify immune cell infiltration between healthy individuals and patients with SCM. The proportions of both innate and acquired immune cell populations increased significantly in patients with SCM. In clinical settings, patients with sepsis are distinguished by an overactive immune response during the initial stage. Although the innate immune response predominates in the early stages of sepsis, innate and adaptive immune responses work together in later stages to reduce the risk of immune suppression and ultimately restore immune homeostasis [57–59]. In our study, we also discovered the highly effective synergistic combination of innate and acquired cell subgroups, including memory B cell and macrophage, immature dendritic cell, and gamma delta T cell.

The mitochondrial function can significantly affect the fate and function of immune cells. The result of correlation analysis revealed that BCS1L, LYRM7, MRPS31, and TIMMDC1 were negatively correlated with activated CD4 T cells, immature dendritic cells, and plasmacytoid dendritic cells. Moreover, FBXO7 and PGS1 were positively associated with activated CD4 T cells, activated CD8 T cells, dendritic cells, monocyte, and neutrophil. These observations support our findings that both innate and acquired immunity are active in patients with SCM. Consequently, the findings of this study also contribute to a better understanding of the relationship between mitochondrial metabolism and immune cells in SCM.

Serial endomyocardial sampling for monitoring SCM process is not feasible. In clinical research, however, it is convenient to measure biomarkers using peripheral blood samples. The single-cell transcriptomic dataset, GSE167363, containing PBMCs from patients with sepsis enabled investigation at the single-cell resolution. Hub gene expression was analyzed in different subsets of PBMCs by the UMAP method. Upregulated genes, such as FBXO7, appeared to be the promising peripheral biomarkers of SCM, which are differentially expressed across a series of cell subpopulations (B cell, CD14^+^ monocyte, and plasma cell) in the peripheral blood of patients with sepsis. We further performed single-cell rank-based GSEA with irGSEA package and visualized the distributions of mitochondria/inflammation-related pathways. These pathways of interest are remarkably enriched in the monocyte and B cell subpopulations, consistent with our above results. This result indicates that alterations in hub gene expression in the monocyte and B cell subpopulation can be used to infer the risk of SCM among patients with sepsis.

Based on the screened mitoDEGs, metformin was identified as the most promising therapeutic agent effective against SCM. More importantly, FBXO7, which was remarkably upregulated in myocardial tissue and peripheral blood, showed the highest binding energy with metformin. These findings were validated in LPS-induced cellular/animal models. The expression of the six hub genes, including TIMMDC1, MRPS31, FBXO7, PGS1, LYRM7, and BCS1L, was determined by qRT-PCR analysis, which exhibited a consistent expression pattern in line with our bioinformatics analysis. Metformin is a classical antidiabetic medication that activates the AMPK pathway and restores mitochondrial metabolism balance [60]. Metformin has various cardioprotective properties. Current studies have demonstrated that a protective role of metformin exerts a protective effect against SCM by downregulating the levels of proinflammatory factors, interacting with IRF4 as well as correcting the bioenergetics imbalance [61, 62]. Seahorse analysis showed that basal respiration, maximum respiration, spare respiratory capacity, and ATP production were all impaired in response to LPS stimulation, but these effects were ameliorated in the metformin treatment group. Metformin’s efficacy was also observed in a mouse model of SCM. Metformin treatment was also found to improve heart function impaired in the SCM group. Metformin could reverse the decrease in mitochondrial membrane potential observed in the SCM group. The hyperactive inflammatory response in the SCM model was relieved following treatment with metformin. Further molecular biology experiments and gene perturbation studies suggest that BCS1L may be a potential target for metformin intervention in SCM. The expression of BCS1L in cardiomyocytes was suppressed in SCM but improved following metformin intervention. Moreover, in BCS1L knockdown models, a diminished ameliorative effect of metformin on SCM was observed, reflected in aspects of mitochondrial metabolism and inflammatory states in cardiomyocytes. These observations underscore the pivotal role of BCS1L in the modulation of mitochondrial function and inflammation in SCM and highlight its potential as a therapeutic target in metformin intervention.

In our study, the crosstalk between mitochondrial metabolism and the immune microenvironment was explored for the first time through bioinformatics analysis of SCM-related datasets. The identification and validation of TIMMDC1, MRPS31, FBXO7, PGS1, LYRM7, and BCS1L as potential molecular targets provide evidence for further investigation of immunometabolism during SCM. As demonstrated by drug prediction and biological experimentation, metformin has potential therapeutic value for the treatment of SCM, as it can restore mitochondrial metabolism and immune homeostasis. However, some limitations of the study warrant further consideration. First, although we validated the hub gene expression in mice, further experiments using human samples are still required to confirm our findings due to the inherent limitations of bioinformatics techniques. Second, since our data were sourced from a database, common covariates such as age, sex, race, and comorbidities were not considered. Further clinical studies and higher levels of evidence are still needed.

## Conclusions

We demonstrated variations in mitochondrial-related genes and immune cell infiltration between SCM and healthy controls through a comprehensive bioinformatics analysis, uncovering the interaction between mitochondrial metabolism and immune infiltration in SCM for the first time. We screened six mitochondria-related candidate hub genes (TIMMDC1, MRPS31, FBXO7, PGS1, LYRM7, and BCS1L) using machine learning algorithms and developed a nomogram for early diagnosis and monitoring of patients with SCM. We validated the therapeutic value of metformin in SCM by combining drug prediction analysis with basic experiments. Metformin significantly improved cardiac function by rebalancing mitochondrial metabolism and immune homeostasis in patients with SCM.

### Supplementary Information

**Additional file 1: Figure S1.** Identification and Functional Analysis of MitoDEGs in SCM. **A** Venn diagram of key module genes versus differentially expressed genes. **B** Venn diagram of mitochondria-related genes versus DEGs. **C** DO analysis. **D** GO analysis. **E**, **F** KEGG analysis. **Figure S2.** Expression analysis of hub genes. **A** Correlation between hub genes. **B**–**G** Expression of six hub genes in SCM and control groups. **Figure S3.** Correlation between hallmark pathways and hub genes. **A**–**F** GSEA analysis of hub genes. Top 5 GSEA enrichment in the high and low expression group of **A** BCS1L, **B** LYRM7, **C** MRPS31, **D** TIMMDC1, **E** FBXO7, **F** PGS1. **G** Correlation between hub genes and hallmark pathways. *p < 0.05, **p < 0.01, ***p < 0.001. **Figure S4.** Infiltration of immune cell types compared between SCM and CON. **A** Heatmap of the proportions of 28 immune cell types; **B** The boxplot of the immune cell proportions; **C** The correlation matrix of immune cell proportions. *p < 0.05, **p < 0.01, ***p < 0.001.

**Additional file 2: Table S1.** Differentially expressed genes between healthy control individuals and patients with SCM.

**Additional file 3: Table S2.** Genelist of each module in Weighted Correlation Network Analysis (WGCNA) analysis.

**Additional file 4: Table S3.** Genelist identified in the support vector machine-recursive feature elimination (SVM-RFE) algorithm.

**Additional file 5: Table S4.** Genelist identified in the least absolute shrinkage and selection operator (LASSO) regression analysis.

**Additional file 6: Table S5.** Genelist identified in the random forest algorithm.

## Data Availability

Datasets supporting the conclusions of this article are available in the NCBI-GEO database (https://www.ncbi.nlm.nih.gov/geo/). All codes are available on GitHub (GitHub, Inc., San Francisco, California) at https://github.com/Lawrence-Albert/SCM_bioinform.

## Abbreviations

SCM: Septic cardiomyopathy
GEO: Gene Expression Omnibus
DEGs: Differentially expressed genes
WGCNA: Weighted correlation network analysis
DSigDB: Drug signatures database
ATP: Adenosine triphosphate
DAMPs: Damage-associated molecular patterns
mPTP: Mitochondrial permeability transition pore
PBMCs: Peripheral blood mononuclear cells
GSEA: Gene Set Enrichment Analysis
GO: Gene Ontology
MF: Molecular functions
BP: Biological pathways
CC: Cellular components
KEGG: Kyoto Encyclopedia of Genes and Genomes
MM: Module membership
GS: Gene significance
SVM: Support vector machine
SVM-RFE: SVM–recursive feature elimination
LASSO: Least Absolute Shrinkage and Selection Operator
ROC: Receiver operating characteristic
AUC: Area under the curve
ssGSEA: Single-sample gene set enrichment analysis
LVEF: Left ventricular ejection fraction
FS: Fraction shortening
LVDd: Left ventricular internal diameter end diastole
LVDs: Left ventricular internal diameter end systole
GTEx: Genotype-Tissue Expression
UMAP: Unified manifold approximation and projection
MD: Molecular docking
