# Supplementary material for: New insights into the role of mitochondrial metabolic dysregulation and immune infiltration in septic cardiomyopathy by integrated bioinformatics analysis and experimental validation
Source: Cell Mol Biol Lett. 2024 Jan 30;29:21. doi: 10.1186/s11658-024-00536-2 (PMC10826082; doi:10.1186/s11658-024-00536-2)
Supplement: Supplementary file 1 — Additional file 1: Figure S1. Identification and Functional Analysis of MitoDEGs in SCM. A Venn diagram of key module genes versus differentially expressed genes. B Venn diagram of mitochondria-related genes versus DEGs. C DO analysis. D GO analysis. E, F KEGG analysis. Figure S2. Expression analysis of hub genes. A Correlation between hub genes. B–G Expression of six hub genes in SCM and control groups. Figure S3. Correlation between hallmark pathways and hub genes. A–F GSEA analysis of hub genes. Top 5 GSEA enrichment in the high and low expression group of A BCS1L, B LYRM7, C MRPS31, D TIMMDC1, E FBXO7, F PGS1. G Correlation between hub genes and hallmark pathways. *p < 0.05, **p < 0.01, ***p < 0.001. Figure S4. Infiltration of immune cell types compared between SCM and CON. A Heatmap of the proportions of 28 immune cell types; B The boxplot of the immune cell proportions; C The correlation matrix of immune cell proportions. *p < 0.05, **p < 0.01, ***p < 0.001. [file 11658_2024_536_MOESM1_ESM.pdf]

Figure S1

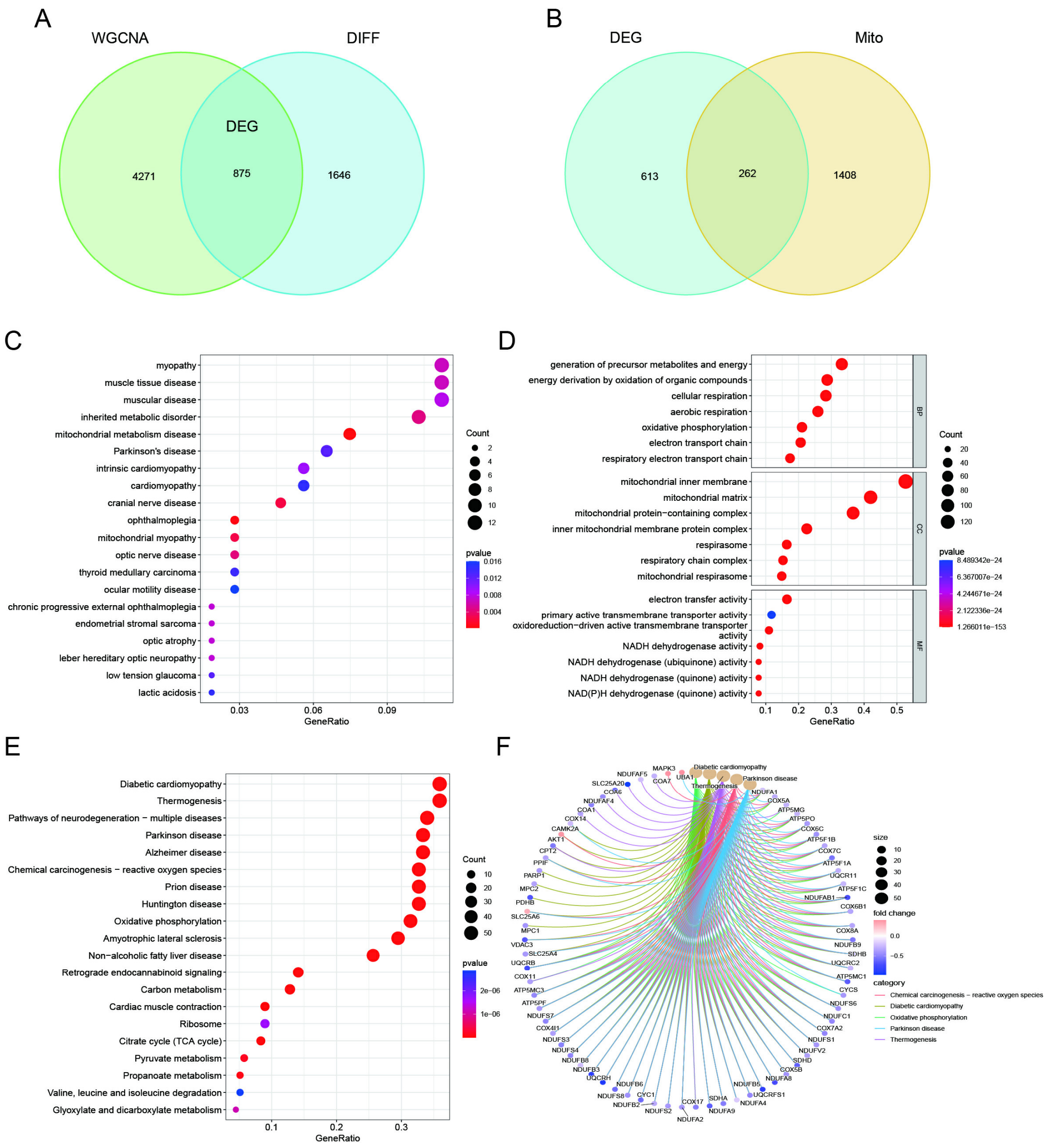

Figure S1. Identification and Functional Analysis of MitoDEGs in SCM.

(A) Venn diagram of key module genes versus differentially expressed genes. (B) Venn diagram of mitochondria-related genes versus DEGs. (C)DO analysis. (D) GO analysis. (E-F) KEGG analysis

Figure S2

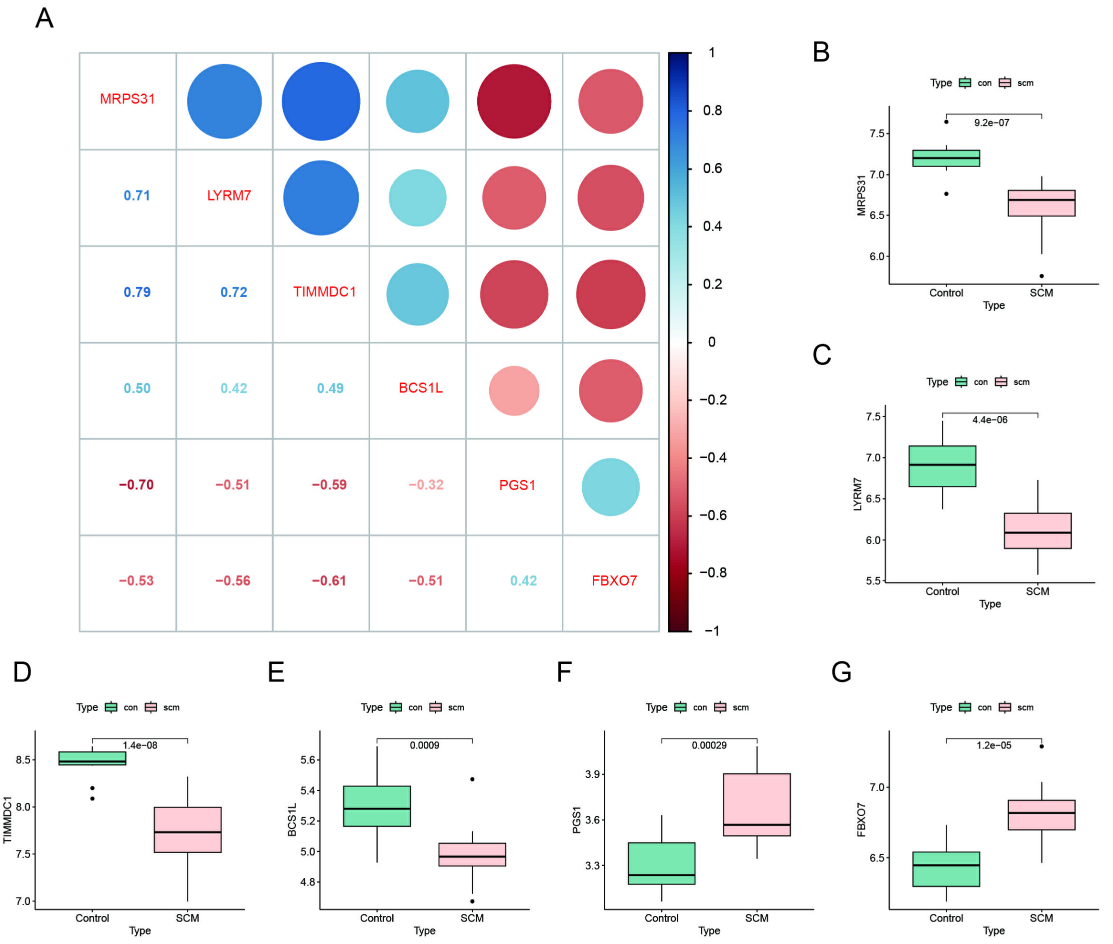

Figure S2. Expression analysis of hub genes.

(A) Correlation between hub genes. (B-G) Expression of six hub genes in SCM and control groups.

Figure S3

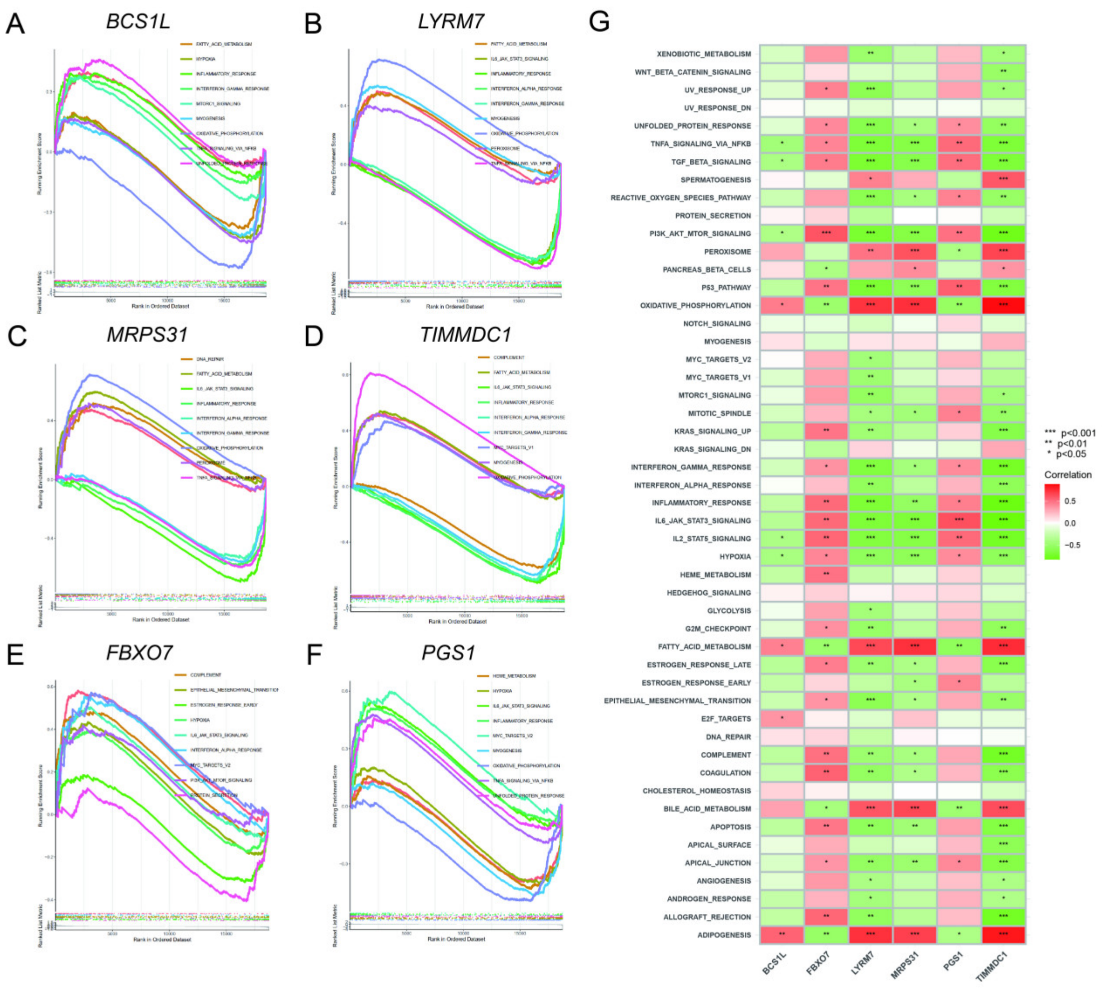

Figure S3. Correlation between hallmark pathways and hub genes.

(A-F) GSEA analysis of hub genes. Top 5 GSEA enrichment in the high and low expression group of (A) BCS1L, (B) LYRM7, (C) MRPS31, (D) TIMMDC1, (E) FBXO7, (F) PGS1. (G) Correlation between hub genes and hallmark pathways. *p < 0.05, **p < 0.01, ***p < 0.001.

Figure S4

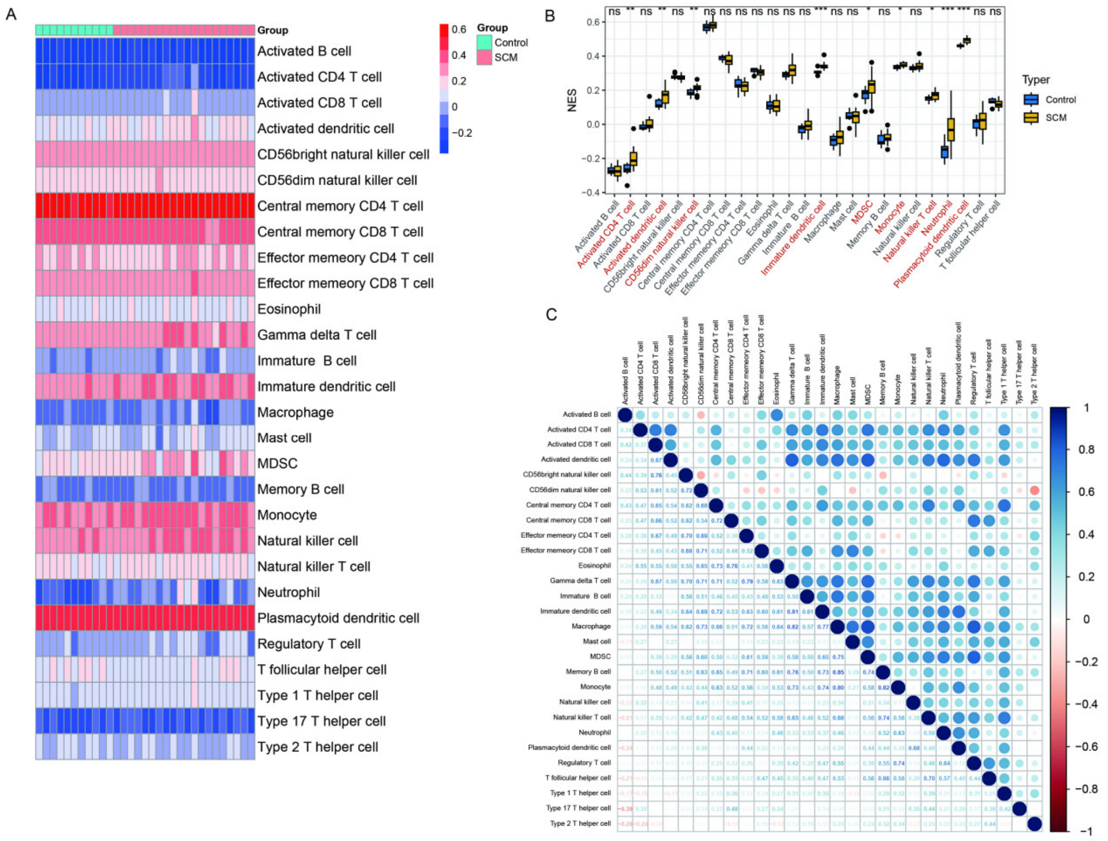

Figure S4. Infiltration of immune cell types compared between SCM and CON.

(A) Heatmap of the proportions of 28 immune cell types; (B) The boxplot of the immune cell proportions; (C) The correlation matrix of immune cell proportions. *p < 0.05, **p < 0.01, ***p < 0.001.
